# Supplementary material for: Dark microbiome and extremely low organics in Atacama fossil delta unveil Mars life detection limits
Source: Nat Commun. 2023 Feb 21;14:808. doi: 10.1038/s41467-023-36172-1 (PMC9944251; doi:10.1038/s41467-023-36172-1)
Supplement: Supplementary file 1 — Supplementary Information file [file 41467_2023_36172_MOESM1_ESM.pdf]

Supplementary Information for: Dark microbiome and extremely low organics in Atacama fossil delta unveil Mars life detection limits

Authors

Armando Azua-Bustos[1,2*], Alberto G. Fairén[1,3], Carlos González-Silva[4], Olga Prieto-Ballesteros[1], Daniel Carrizo[1], Laura Sánchez-García[1], Victor Parro[1], Miguel Ángel Fernández-Martínez[5], Cristina Escudero[1], Victoria Muñoz-Iglesias[1], Maite Fernández-Sampedro[1], Antonio Molina[1], Miriam García Villadangos[1], Mercedes Moreno-Paz[1], Jacek Wierzchos[6], Carmen Ascaso[6], Teresa Fornaro[7], John Robert Brucato[7], Giovanni Poggiali[7], Jose Antonio Manrique[8,9], Marco Veneranda[8], Guillermo López-Reyes[8], Aurelio Sanz-Arranz[8], Fernando Rull[8], Ann M. Ollila[10], Roger C. Wiens[10], Adriana Reyes-Newell[11], Samuel M. Clegg[10], Maëva Millan[12,13,14], Sarah Stewart Johnson[12,15], Ophélie McIntosh[7,15], Cyril Szopa[15], Caroline Freissinet[15], Yasuhito Sekine[16,17], Keisuke Fukushi[17], Koki Morida[18], Kosuke Inoue[18], Hiroshi Sakuma[19], and Elizabeth Rampe[20].

Affiliations

[1]Centro de Astrobiología (CSIC-INTA), 28850 Madrid, Spain.

[2]Instituto de Ciencias Biomédicas, Facultad de Ciencias de la Salud, Universidad Autónoma de Chile, Santiago, Chile.

[3]Department of Astronomy, Cornell University, Ithaca, 14853 NY, USA.

[4]Facultad de Ciencias, Universidad de Tarapacá, Chile.

[5]Department of Ecology, Universidad Autónoma de Madrid, 28049 Madrid, Spain

[6]Museo Nacional de Ciencias Naturales (CSIC), 28006 Madrid, Spain.

[7]INAF-Astrophysical Observatory of Arcetri, Florence, Italy.

[8]Universidad de Valladolid, Valladolid, Spain.

[9] Institut de Recherche en Astrophysique et Planétologie (IRAP) Toulouse, France.

[10] Purdue University, Earth, Atmospheric, and Planetary Sciences, USA.

[11] Southwest Sciences, Inc. 1570 Pacheco St. Ste. E11. Santa Fe, NM. 87505. USA.

[12]Department of Biology, Georgetown University, Washington DC 20057, USA.

[13]NASA Goddard Space Flight Center, Solar System Exploration Division, Greenbelt, MD 20771, USA.

[14]LATMOS/IPSL, UVSQ Université Paris-Saclay, Sorbonne Université, CNRS, 11 Bd d'Alembert, 78280 Guyancourt, France.

[15]Science, Technology, and International Affairs Program, Georgetown University, Washington DC 20057, USA.

[16]Earth-Life Science Institute (ELSI), Tokyo Institute of Technology, Japan.

[17]Institute of Nature and Environmental Technology, Kanazawa University, Japan.

[18]Division of Natural System, Kanazawa University, Japan.

[19]National Institute for Materials Science, Japan.

[20]Astromaterials Research and Exploration Science Division, NASA Johnson Space Center, Houston, TX, US.

*Corresponding author: Armando Azua-Bustos (aazua@cab.inta-csic.es)

Supporting Information

Mineralogical testbed instrument results (DRIFTS, LIBS, RLS)

Materials and Methods

Supplementary Tables and Figures

Tables S1 to S5

Figs. S1 to S18

Total Ion Chromatograms (TIC)

Supplementary References

Mineralogical testbed instrument results (DRIFTS, LIBS, RLS)

DRIFTS (Diffuse Reflectance Infrared Fourier Transform Spectroscopy) analyses carried out in the Near InfraRed (NIR) spectral range of instruments on board a number of missions, such as the NASA Mars 2020 rover mission like SuperCam, and on board the ESA-Roscosmos ExoMars 2022 rover mission like Ma_MISS

(Mars Multispectral Imager for Subsurface Studies), ISEM (Infrared Spectrometer for ExoMars), and MicrOmega (also ExoMars), showed similar mineral infrared features for all samples. The strongest features observed in the NIR spectral region show the presence of analcime, calcite, hematite, albite, and minor peaks indicating the presence of vermiculite (fig. S11A). The consistency between XRD and NIR data confirms that this latter technique, employed by the Mars 2020 Perseverance rover and to be employed by the ExoMars Rosalind Franklin rover is indeed the prime method for mineral identification.

In turn, when the unsupervised automatic operation simulator of RLS (Raman Laser Spectrometer)[1], onboard the ExoMars rover was used[2,3], most of the aforementioned minerals at different concentrations were detected, (Table S4), although the detection of phyllosilicates was highly impaired by the higher noise and background, while low concentrations minerals such as perchlorates were not easily detected. When samples were crushed and sieved to a granulometry similar to that of the crusher onboard the rover Rosalind Franklin rover of the ExoMars mission[4] and then studied with human operated top bench instruments such as Fourier Transform Raman spectrometer and Micro Raman for individual grain analyses, the presence of hematite, vermiculite, calcite, sodium rich-feldspar, calcium sulfate (in different states of hydration) and quartz were confirmed as mayor components of these samples (Figure S12 and Table S3 and S4). The presence of anatase (TiO2) monocrystals is of interest, as titanium oxides have been suggested as very ancient biosignatures[5]. A single crystal of sodium perchlorate was also detected using Micro Raman in LZ samples, a compound of importance detected on Mars[6], due to its role as an strong oxidant of organic species[7].

LIBS (Laser Induced Breakdown Spectroscopy) analyses of these samples, obtained by a laboratory replica of ChemCam onboard the Curiosity rover, (which uses a powerful laser pulse to ablate atoms and ions in electronically excited states[8,9]), detected that relative to the other samples, LZ samples were enriched in Ti (fig. S13A), and that all but the evaporite samples appear enriched in FeOT (11-14 wt%), while silicon appears higher in U1, and calcium enriched in LZ samples (fig. S13B). Evaporite samples were enriched in sodium (fig. S13C), and the Na/K ratio of evaporite samples was high, as potassium was at least with this instrument,

hardly detected. Similarly, although LIBS does not detect Cl easily, the findings of this instrument suggests that sodium was in the form of NaCl, in agreement with XRD analyses.

Supplementary Tables and Figures

Supplementary Tables S1 to S5

Table S1. Identification of bacterial sequences obtained by Next-Generation Sequencing (NGS). Numbers are percentages of total sequences.

Species	Sandstones	Evaporites	Lower Regolith	Upper Regolith	U1	U2
Acinetobacter johnsonii	-	-	-	-	0,18	-
Acinetobacter sp.	-	0,27	-	-	-	-
Aquisalimonas halophila	-	-	-	0,04	-	-
Arthrobacter pascens	0,17	-	-	-	-	-
Arthrobacter sp1	-	2,35	-	-	-	-
Arthrobacter sp2	-	0,36	-	0,02	0,29	-
Arthrobacter sp3	-	-	-	0,23	-	-
Arthrobacter sp4	0,97	-	-	-	-	-
Asticcacaulis sp	-	-	-	0,03	-	-
Bacillus sp1	0,20	-	-	-	-	-
Bacillus sp2	0,20	-	-	-	-	-
Bacillus sp3	0,15	-	-	-	-	-
Bacillus sp4	-	-	-	0,36	-	-
Bacillus sp5	-	0,03	-	1,13	-	0,18
Bacillus sp6	-	-	-	0,04	-	-
Bacillus sp7	-	-	-	0,82	-	-
Bacillus sp8	-	-	-	0,22	-	-
Bacillus sp9	-	0,17	-	-	-	-
Bacillus sp10	-	-	-	0,16	-	-
Bacillus sp11	-	-	-	0,10	-	-
Bacillus sp12	-	-	-	-	0,05	0,03
Bacillus sp13	-	-	-	0,07	-	-
Bacillus sp14	-	-	-	0,06	-	-
Bacteroides sp.	-	-	-	-	0,71	-
Blastococcus sp1	-	-	-	1,39	-	0,16
Blastococcus sp2	-	-	-	1,12	-	-
Blastococcus sp3	0,61	-	-	-	-	-
Blastococcus sp4	0,58	-	-	-	-	-
Blastococcus sp5	0,46	-	-	-	-	-
Blastococcus sp6	0,45	-	-	-	-	-
Blastococcus sp7	0,42	-	-	-	-	-
Blastococcus sp8	0,34	-	-	-	-	-
Blastococcus sp9	0,21	-	-	-	-	-
Blastococcus sp10	0,16	-	-	-	-	-
Bradyrhizobium sp1	-	-	-	0,02	-	-
Bradyrhizobium sp2	0,42	-	-	-	-	-
Brevibacillus sp.	-	0,13	-	-	-	-
Comamonas aquatica	-	0,10	-	-	-	-
Corynebacterium sp1	-	0,37	0,55	0,07	0,68	0,30
Corynebacterium sp2	0,21	-	-	-	-	-
Corynebacterium sp3	-	0,34	-	0,03	-	0,12
Corynebacterium sp4	-	-	-	-	-	0,03
Corynebacterium sp5	0,10	-	-	-	-	-
Corynebacterium sp6	-	-	-	-	-	0,03
Deinococcus sp.	-	0,23	-	-	-	-
Delftia sp.	0,17	-	-	-	-	-
Desulfosporosinus sp.	-	-	-	0,23	-	-
Desulfovibrio sp.	-	-	-	-	0,44	-
Eubacteria sp.	-	-	-	0,05	-	-
Halanaerobium sp.	-	-	-	0,18	-	-
Hydrogenophilus sp.	-	-	-	-	0,05	-
Hymenobacter sp	-	-	-	-	0,09	-
Kocuria sp.	-	0,16	-	-	-	-
Lysinibacillus sp1	-	-	0,07	-	-	-
Lysinibacillus sp2	-	-	3,83	-	-	-
Massilia sp1	-	0,11	-	0,05	0,13	-
Massilia sp2	-	-	-	-	-	0,05
Massilia sp3	-	-	-	-	0,25	-
Methylobacterium sp.	-	0,26	-	-	-	-
Methylorubrum sp.	-	0,04	-	-	-	-
Methyloversatilis sp.	0,11	-	-	-	-	-
Nocardioides sp1	-	-	-	0,36	-	-
Nocardioides sp2	-	0,20	-	0,11	-	-
Nocardioides sp3	-	-	-	0,14	-	-
Paenibacillus sp1	-	-	-	0,18	-	-
Paenibacillus sp2	-	-	-	0,10	-	-
Paenibacillus sp3	-	-	-	0,07	-	-
Paenibacillus sp4	-	-	-	0,05	-	-
Paenibacillus sp5	-	-	-	0,02	-	-
Paenibacillus sp6	-	-	-	0,03	-	-
Pantoea sp.	-	-	-	-	0,52	-
Pedobacter sp.	-	-	-	-	-	0,03
Phyllobacterium sp.	-	14,66	9,43	2,35	6,94	4,22
Pontibacter sp.	0,37	-	-	-	-	-
Pseudomonas sp1	-	2,36	-	-	-	-
Pseudomonas sp2	-	-	0,43	-	-	-
Pseudomonas sp3	-	-	-	0,07	-	-
Ralstonia sp1	-	0,49	0,05	-	-	-
Ralstonia sp2	-	0,16	0,32	-	-	-
Ramlibacter sp.	-	-	-	0,02	-	-
Rhodobaca sp.	-	-	-	0,12	-	-
Roseomonas sp.	0,09	-	-	-	-	-
Rubrobacter sp.	-	-	-	-	0,13	-
Rubrobacterineae sp.	0,57	-	-	-	-	-
Saccharibacteria sp1	0,37	-	-	-	-	-
Saccharibacteria sp2	0,27	-	-	-	-	-
Sphingobium sp.	-	-	-	-	0,71	-
Sphingomonas sp1	-	1,99	-	0,01	0,17	-
Sphingomonas sp2	-	-	-	0,12	-	-
Sphingomonas sp3	-	-	-	0,22	-	-
Thermoactinomyces sp.	-	-	-	-	-	0,26
Thermomonas sp.	-	-	-	0,03	-	-
Unc. *Acidibacter* sp1	0,24	-	-	-	-	-
Unc. *Acidibacter* sp2	0,24	-	-	-	-	-
Unc. Acidimicrobidae	1,10	-	-	-	-	-
Unc. Acidimicrobineae	0,90	-	-	-	-	-
Unc. *Acidimicrobium* sp.	-	-	-	0,02	-	-
Unc. *Acidisphaera* sp.	-	-	-	0,11	-	-
Unc. Acidobacteria sp.	-	-	-	0,07	-	-
Unc. Actinobacteria sp1	-	0,10	-	-	-	-
Unc. Actinobacteria sp2	-	-	-	0,05	-	-
Unc. Actinobacteria sp3	-	-	-	0,03	-	-
Unc. Bacillaceae	-	-	-	0,07	-	-
Unc. Bacteroidetes	-	0,54	-	-	-	-
Unc. *Bdellovibrio* sp.	0,02	-	-	-	-	-
Unc. Burkholderiaceae	-	-	-	-	0,05	-
Unc. Burkholderiales	0,23	-	-	-	-	-
Unc. *Caulobacter* sp1	-	-	-	0,04	-	-
Unc. *Caulobacter* sp2	-	-	-	0,03	-	-
Unc. *Chloroflexota* sp1	-	-	-	0,10	-	-
Unc. *Chloroflexota* sp2	-	-	-	0,06	-	-
Unc. *Chloroflexota* sp3	-	-	-	0,03	-	-
Unc. Euzebyaceae	0,18	-	-	-	-	-
Unc. Euzebyales sp1	0,49	-	-	-	-	-
Unc. Euzebyales sp2	0,36	-	-	-	-	-
Unc. Euzebyales sp3	0,20	-	-	-	-	-
Unc. Euzebyales sp4	0,17	-	-	-	-	-
Unc. *Ferrimicrobium* sp1	0,20	-	-	-	-	-
Unc. *Ferrimicrobium* sp2	0,19	-	-	-	-	-
Unc. Frankineae	1,00	-	-	-	-	-
Unc. Gemmatimonadetes sp1	0,48	-	-	-	-	-
Unc. Gemmatimonadetes sp2	0,46	-	-	-	-	-
Unc. Gemmatimonadetes sp3	0,15	-	-	-	-	-
Unc. Gemmatimonadetes sp4	-	-	-	0,08	-	-
Unc. Gemmatimonadetes sp5	-	-	-	0,03	-	-
Unc. Gemmatimonadetes sp6	-	-	-	0,02	-	-
Unc. Gemmatimonadetes sp7	-	-	-	0,03	-	-
Unc. Gemmatimonadetes sp8	-	-	-	0,03	-	-
Unc. *Gemmatimonas* sp1	-	-	-	0,13	-	-
Unc. *Gemmatimonas* sp2	-	-	-	0,29	-	-
Unc. *Marmoricola* sp1	-	-	-	-	0,10	-
Unc. *Marmoricola* sp2	0,14	-	-	-	-	-
Unc. Micrococcineae sp1	-	-	-	1,08	0,30	-
Unc. Micrococcineae sp2	1,19	-	-	-	-	-
Unc. Planctomycetota sp1	-	-	-	0,15	-	-
Unc. Planctomycetota sp2	-	-	-	0,12	-	-
Unc. *Pseudonocardia* sp1	0,31	-	-	-	-	-
Unc. *Pseudonocardia* sp2	0,16	-	-	-	-	-
Unc. Rhizobiales sp1	-	-	-	0,06	-	-
Unc. *Rubrobacter* sp.	-	-	-	0,10	-	-
Unc. Rubrobacteraceae	-	-	-	0,07	-	-
Unc. *Solirubrobacter* sp1	0,17	-	-	-	-	-
Unc. *Solirubrobacter* sp2	-	-	-	-	0,16	-
Unc. *Solirubrobacter* sp3	0,15	-	-	-	-	-
Unc. *Solirubrobacter* sp4	0,21	-	-	-	-	-
Unc. Solirubrobacterales sp1	1,14	-	-	-	-	-
Unc. Solirubrobacterales sp2	0,37	-	-	-	-	-
Unc. Solirubrobacterales sp3	0,24	-	-	-	-	-
Unc. Solirubrobacterales sp4	0,31	-	-	-	-	-
Unc. Solirubrobacterales sp5	0,21	-	-	-	-	-
Unc. Solirubrobacterales sp6	0,19	-	-	-	-	-
Unc. Solirubrobacterales sp7	-	-	-	0,04	-	-
Unc. Sphingobacteriales sp1	-	-	-	0,13	-	-
Unc. Sphingobacteriales sp2	-	-	-	0,55	-	-
Unc. *Sporichthya* sp1	0,35	-	-	-	-	-
Unc. *Sporichthya* sp2	0,18	-	-	-	-	-
Unc. *Sporichthya* sp3	0,17	-	-	-	-	-
Unclassified	-	-	-	1,04	-	-
Unclassified	-	0,39	-	-	-	-
Unclassified	-	-	-	0,21	-	-
Unclassified	-	-	-	-	-	0,21
Unclassified	-	-	-	0,15	-	-
Unclassified	-	0,91	-	-	-	-
Unclassified	-	-	-	-	0,73	-
Unclassified	-	0,73	-	-	-	-
Unclassified	-	-	0,71	-	-	-
Unclassified	-	-	-	-	0,71	-
Unclassified	-	0,19	0,13	0,09	0,10	0,11
Unclassified	-	0,40	0,18	-	-	-
Unclassified	-	-	-	-	-	0,15
Unclassified	-	-	-	0,13	-	-
Unclassified	-	0,12	-	-	-	-
Unclassified	-	-	-	0,11	-	-
Unclassified	-	0,10	-	-	-	-
Unclassified	-	-	-	0,07	-	-
Unclassified	-	-	-	0,05	-	-
Unclassified	-	-	-	0,21	0,74	-
Unclassified	-	-	-	0,04	-	-
Unclassified	-	-	-	0,04	-	-
Unclassified	-	0,04	-	-	-	-
Unclassified	-	0,03	-	-	-	-
Unclassified	-	-	-	0,03	-	-
Unclassified	-	-	-	0,03	-	-
Unclassified	-	-	-	0,03	-	-
Unclassified	-	-	-	0,02	-	-
Unclassified	-	-	-	0,02	-	-

Table S2. Colony Forming Units (CFU) per gram of sample type.

	Upper Regolith	U1	U2	Sandstones	Evaporites	Lower Regolith
CFU/g	$8.0x10^1$	$3.0x10^1$	$1.0x10^1$	$9.0x10^1$	$1.1x10^1$	$2.0x10^1$

Table S3. Mineral phases found by a human operated micro Raman at 633 nm. M: Major phase) D: Detected as individual grains. DHS: different states of hydration. nd: not detected.

Mineral Phase	Upper Zone	Lower Zone	Sandstones	Evaporites	U1	U2
Hematite	M	M	M	M	M	M
Anatase	M	M	M	M	M	M
Quartz	M	M	M	M	M	M
Feldspars	M	M	M	M	M	M
Analcime	nd	nd	nd	nd	nd	D
Phyllosilicates	M	D	D	M	M	M
Calcite	M	M	M	M	M	M
Calcium sulfate	DHS	DHS	DHS	DHS	nd	DHS
Amphibole	D	D	D	D	nd	nd
Epidote	D	D	D	nd	nd	D
Apatite	D	nd	nd	nd	D	D
Pyroxene	D	nd	nd	nd	nd	D
Perchlorate	nd	D	nd	nd	nd	nd

Table S4. Mineral phases found by unsupervised RLS operation simulator mode (100 spots). M: Major phase (more than 75% of the analyzed spots), nd: not detected.

Mineral Phase		Upper Zone	Lower Zone	Sandstones	Evaporites	U1	U2
Hematite		M	M	M	M	M	M
Anatase		M	M	M	M	M	M
Quartz		M	M	M	M	M	M
Feldspars	Orthose	M	M	nd	M	M	M
	High Na	M	M	U	M	M	M
Analcime		<5	<10	nd	nd	<5	<10
Vermiculite		<10	<10	<10	>10	nd	<10
Calcite		M	M	M	M	M	M
Calcium sulfa	Gypsum	nd	<10	1	High Content (mixed)	<10	nd
	Mixed hydration	<10	<10	2	nd	nd	nd
	Anhydrite	<10	nd	>10	nd	<10	<10
Diopside		<5	<5	nd	nd	nd	<5
Epidote		nd	1	nd	<10	<10	1
Apatite		2	nd	nd	1	1	<10
Dolomite		nd	nd	nd	1	nd	<10

Table S5. Red Stone (infra-red) IR Organic assignments

Upper Zone			Lower Zone			Sandstones		
cm^{-1}	I	Assignment*	cm^{-1}	I	Assignment*	cm^{-1}	I	Assignment*
**14650**	broad	Hematite (15000)?	**14650**	broad	Hematite (15000)?	**14650**	broad	Hematite (15000)?
**11550**	broad	Hematite (11550) / Vermiculite (11550)	**11550**	broad	Hematite (11550) / Vermiculite (11550)	**11550**	broad	Hematite (11550) / Vermiculite (11550)
**7343**	w	Organics (second overtone of the C-H stretching mode or combination band)?	**7343**	mw	Organics (second overtone of the C-H stretching mode or combination band)?	**7343**	w	Organics (second overtone of the C-H stretching mode or combination band)?
**7050**	m	Albite (7092)	**7170**	w	Vermiculite (7168)	**7170**	w	Vermiculite (7168)
**5233**	ms	Albite (5236)	**5233**	w	Albite (5236)	**5233**	w	Albite (5236)
**4534**	m	Vermiculite (4525) / Albite (4545)	**4534**	w	Vermiculite (4525) / Albite (4545)			
**4445**	vw	Organics (first overtone of the C–H stretching mode)?	**4444**	w	Organics (first overtone of the C–H stretching mode)?	**4450**	vw	Organics (first overtone of the C–H stretching mode)?
**4357**	vw	Vermiculite (4330)?	**4357**	vw	Vermiculite (4330)?	**4357**	vw	Vermiculite (4330)?
**4280**	m broad	Analcime (4275)	**4280**	m broad	Analcime (4275)	**4313**	vw broad	Analcime (4275)?
**4180**	vw	Vermiculite (4180)	**4180**	vw	Vermiculite (4180)	**4180**	vw	Vermiculite (4180)
**3930**	w	Calcite (3940)	**3930**	w	Calcite (3940)	**3930**	w	Calcite (3940)
**3678**	vw	Vermiculite (3696)?	**3672**	vw	Vermiculite (3696)?	**3672**	vw	Vermiculite (3696)?
**3585**	s broad	Calcite (3584) / Hematite shape (3550)	**3580**	s broad	Calcite (3584) / Hematite shape (3550)	**3580**	s broad	Calcite (3584) / Hematite shape (3550)
**3410**	ms	Hematite (3410)	**3420**	ms	Hematite (3410)	**3420**	ms	Hematite (3410)
**2982**	m	Calcite (2980) (CH stretchings covered by strong mineral peak)	**2982**	s	Calcite (2980) (CH stretchings covered by strong mineral peak)	**2982**	vw	Calcite (2980) (CH stretchings covered by strong mineral peak)
**2874**	m	Calcite (2872) (CH stretchings covered by strong mineral peak)	**2874**	s	Calcite (2872) (CH stretchings covered by strong mineral peak)	**2874**	vw	Calcite (2872) (CH stretchings covered by strong mineral peak)
			**2667**	vw	Quartz (2670)			
**2594**	m broad	Quartz (2593)?	**2584**	w broad	Quartz (2593)?	**2600**	w broad	Quartz (2593)?
**2510**	ms	Calcite (2510 s) / Albite (2510 w)	**2510**	s	Calcite (2510 s) / Albite (2510 w)	**2510**	m	Calcite (2510 s) / Albite (2510 w)
**2360**	vw	Hematite (2346)	**2360**	vw	Hematite (2346)	**2360**	vw	Hematite (2346)
			**2230**	w	Gypsum (2230)			
**2135**	w	Quartz (2133) / Calcite (2137)	**2135**	w	Quartz (2133) / Calcite (2137)			
**2090**	vw	Halite (2090)	**2090**	vw	Halite (2090)			
**2030**	vw	Analcime (2030)	**2030**	vw	Analcime (2030)			
			**1995**	m broad	Gypsum (2008)?	**1992**	w broad	Gypsum (2008)?
**1942**	vw	Organics (combination band)?	**1942**	vw	Organics (combination band)?	**1942**	vw	Organics (combination band)?
**1870**	vw	Hematite (1871)	**1867**	ms	Hematite (1871)	**1867**	vw	Hematite (1871)
**1795**	m	Calcite (1795)	**1795**	s	Calcite (1795)	**1795**	w broad	Calcite (1795)
**1769**	vw	Organics (carbonyl stretching)?	**1769**	vw	Organics (carbonyl stretching)?	**1769**	vw	Organics (carbonyl stretching)?
**1747**	vw	Organics (carbonyl stretching)?	**1747**	vw	Organics (carbonyl stretching)?	**1747**	vw	Organics (carbonyl stretching)?
**1732**	vw	Calcite (1730)	**1732**	vw	Calcite (1730)	**1732**	vw	Calcite (1730)
**1717**	vw	Organics (carbonyl stretching)?	**1717**	vw	Organics (carbonyl stretching)?	**1717**	vw	Organics (carbonyl stretching)?
**1700**	vw	Organics (carbonyl stretching)?	**1700**	vw	Organics (carbonyl stretching)?	**1700**	vw	Organics (carbonyl stretching)?
**1684**	vw	Gypsum (1684)	**1684**	w	Gypsum (1684)			
**1653**	vw	Vermiculite (1647)	**1653**	vw	Vermiculite (1647)			
**1628**	ms	Calcite (1625) / Hematite (1625)	**1622**	ms	Calcite (1625) / Hematite (1625) / Gypsum (1620)	**1626**	ms	Calcite (1625) / Hematite (1625) / Gypsum (1620)
			**1587**	vw	Calcite (1588)			
**1560**	vw	Hematite (1562)	**1560**	vw	Hematite (1562)	**1560**	vw	Hematite (1562)
**1522**	vw	Organics (C=C stretching in conjugated systems)?	**1522**	vw	Organics (combination band)?			
**1508**	vw	Organics (C=C stretching in conjugated systems)?	**1508**	vw	Organics (combination band)?	**1508**	vw	Organics (C=C stretching in conjugated systems)?
**1489**	vw	Organics (CH bending)?	**1489**	vw	Organics (CH bending)?	**1489**	vw	Organics (CH bending)?
**1474**	vw	Organics (CH bending)?	**1474**	vw	Organics (CH bending)?			
**1456**	vw	Organics (CH bending)?	**1456**	vw	Organics (CH bending)?	**1456**	vw	Organics (CH bending)?
**1437**	vw	Organics (CH bending)?	**1437**	vw	Organics (CH bending)?	**1437**	vw	Organics (CH bending)?
**1420**	vw	Organics (CH bending)?	**1420**	vw	Organics (CH bending)?	**1420**	vw	Organics (CH bending)?
**1366**	s broad	Calcite (1375) / Halite (1363)	**1385**	s broad	Calcite (1375)	**1340**	s broad	Convolution of many peaks
**1339**	w in the broad peak	Organics (C-O stretching or CH bending)?	**1339**	w in the broad peak	Organics (C-O stretching)?			
**1315**	w in the broad peak	Organics (C-O stretching or CH bending)?	**1315**	w in the broad peak	Organics (C-O stretching)?			
**1281**	m broad	Albite (1277)	**1281**	m broad	Albite (1277)	**1260**	m broad	Albite (1277)
			**1159**	m	Quartz (1157 s) / Hematite (1157)	**1159**	w	Quartz (1157 s) / Hematite (1157)
**1120**	s broad	Possible convolution of Albite, Quartz, Calcite, Vermiculite Peaks	**1115**	s broad	Halite (1109) + Possible convolution of Albite, Quartz, Calcite, Vermiculite Peaks	**1124**	s broad	Halite (1109) + Possible convolution of Albite, Quartz, Calcite, Vermiculite Peaks
			**1060**	m	Calcite (1065)			
**1022**	w	Hematite (1022)	**1022**	w	Hematite (1022)	**1024**	vw	Hematite (1022)
**920**	m broad	Calcite (935)?	**945**	m broad	Calcite (945)	**940**	m broad	Calcite (945)
**870**	w	Calcite (872)	**870**	w	Calcite (872)	**870**	vw	Calcite (872)
**847**	w	Calcite (847)	**847**	w	Calcite (847)	**847**	vw	Calcite (847)
**720**	very broad	Convolution of many peaks	**715**	very broad	Convolution of many peaks with Calcite (712) the most important	**715**	very broad	Convolution of many peaks with Calcite (712) the most important
**635**	n the broad p	Albite (633)	**638**	the broad p	Albite (633)			
			**613**	vw	Halite (619)	**620**	vw broad	Halite (619)
**557**	m broad	Albite (555)	**567**	m broad	Quartz (571)	**567**	m broad	Quartz (571)
**510**	m	Hematite (500) / Quartz (522)	**520**	m	Quartz (522)	**505**	m broad	Hematite (500) / Quartz (522)
**455**	m	Albite (455)	**455**	m	Albite (455)	**455**	m	Albite (455)
			**444**	vw	Calcite (436)			
**422**	vw	Analcime (434)	**434**	vw	Analcime (434)			
			**420**	vw	Albite (420)	**432**	vw	Albite (420)
**403**	m	Quartz (403)	**405**	m	Quartz (403)	**405**	w	Quartz (403)

Table S5. Red Stone (infra-red) IR Organic assignments (continued)

Evaporites			Unit 1			Unit 2		
cm^{-1}	I	Assignment*	cm^{-1}	I	Assignment*	cm^{-1}	I	Assignment*
**14650**	broad	Hematite (15000)?	**14650**	broad	Hematite (15000)?	**14650**	broad	Hematite (15000)?
**11550**	broad	Hematite (11550) / Vermiculite (11550)	**11550**	broad	Hematite (11550) / Vermiculite (11550)	**11550**	broad	Hematite (11550) / Vermiculite (11550)
**9725**	broad	NA						
**7343**	w	Organics (second overtone of the C-H stretching mode or combination band)?	**7343**	mw	Organics (second overtone of the C-H stretching mode or combination band)?	**7343**	mw	Organics (second overtone of the C-H stretching mode or combination band)?
**7170**	w	Vermiculite (7168)	**7100**	m	Albite (7092)	**7100**	w	Albite (7092)
**5233**	m	Albite (5236)	**5223**	w	Analcime (5222)	**5233**	m	Albite (5236)
**4556**	vw	Albite (4545)	**4530**	m	Vermiculite (4525) / Albite (4545)	**4542**	w	Albite (4545)
**4450**	vw	Organics (first overtone of the C–H stretching mode)?	**4440**	mw	Organics (first overtone of the C–H stretching mode)?	**4448**	vw	Organics (first overtone of the C–H stretching mode)?
**4350**	vw	Vermiculite (4330)?	**4357**	vw	Vermiculite (4330)?	**4357**	vw	Vermiculite (4330)?
**4313**	w broad	Analcime (4275)?	**4280**	m broad	Analcime (4275)	**4307**	m broad	Analcime (4275)
**4180**	vw	Vermiculite (4180)	**4180**	vw	Vermiculite (4180)	**4180**	vw	Vermiculite (4180)
**3930**	w	Calcite (3940)	**3930**	w	Calcite (3940)	**3930**	w	Calcite (3940)
**3672**	vw	Vermiculite (3696)?				**3672**	vw	Vermiculite (3696)?
**3580**	s broad	Calcite (3584) / Hematite shape (3550)	**3590**	s broad	Calcite (3584) / Hematite shape (3550)	**3580**	s broad	Calcite (3584) / Hematite shape (3550)
**3420**	ms	Hematite (3410)	**3420**	ms	Hematite (3410)	**3420**	ms	Hematite (3410)
**2982**	ms	Calcite (2980) (CH stretchings covered by strong mineral peak)	**2982**	m	Calcite (2980) (CH stretchings covered by strong mineral peak)	**2982**	m	Calcite (2980) (CH stretchings covered by strong mineral peak)
**2874**	ms	Calcite (2872) (CH stretchings covered by strong mineral peak)	**2874**	m	Calcite (2872) (CH stretchings covered by strong mineral peak)	**2874**	m	Calcite (2872) (CH stretchings covered by strong mineral peak)
**2594**	m broad	Quartz (2593)?	**2594**	m broad	Quartz (2593)?	**2594**	m broad	Quartz (2593)?
**2510**	s	Calcite (2510 s) / Albite (2510 w)	**2510**	ms	Calcite (2510 s) / Albite (2510 w)	**2510**	ms	Calcite (2510 s) / Albite (2510 w)
**2360**	vw	Hematite (2346)	**2360**	vw	Hematite (2346)	**2360**	vw	Hematite (2346)
**2230**	vw	Gypsum (2230)	**2230**	w	Gypsum (2230)	**2230**	vw	Gypsum (2230)
**2131**	w	Quartz (2133) / Calcite (2137)	**2135**	w	Quartz (2133) / Calcite (2137)	**2135**	w	Quartz (2133) / Calcite (2137)
**2090**	vw	Halite (2090)	**2090**	vw	Halite (2090)	**2090**	vw	Halite (2090)
**2030**	vw	Analcime (2030)	**2030**	vw	Analcime (2030)	**2030**	vw	Analcime (2030)
**1992**	w broad	Gypsum (2008)?	**1995**	m broad	Gypsum (2008)?	**1995**	m broad	Gypsum (2008)?
**1942**	vw	Organics (combination band)?	**1942**	vw	Organics (combination band)?	**1942**	vw	Organics (combination band)?
**1867**	w	Hematite (1871)	**1870**	ms	Hematite (1871)	**1870**	w	Hematite (1871)
**1795**	s	Calcite (1795)	**1795**	ms	Calcite (1795)	**1795**	ms	Calcite (1795)
**1769**	vw	Organics (carbonyl stretching)?	**1769**	vw	Organics (carbonyl stretching)?	**1769**	vw	Organics (carbonyl stretching)?
**1747**	vw	Organics (carbonyl stretching)?	**1747**	vw	Organics (carbonyl stretching)?	**1747**	vw	Organics (carbonyl stretching)?
**1732**	vw	Calcite (1730)	**1732**	vw	Calcite (1730)	**1732**	vw	Calcite (1730)
**1717**	vw	Organics (carbonyl stretching)?	**1717**	vw	Organics (carbonyl stretching)?	**1717**	vw	Organics (carbonyl stretching)?
**1700**	vw	Organics (carbonyl stretching)?	**1700**	vw	Organics (carbonyl stretching)?	**1700**	vw	Organics (carbonyl stretching)?
**1684**	w	Gypsum (1684)	**1684**	w	Gypsum (1684)	**1684**	w	Gypsum (1684)
**1653**	vw	Vermiculite (1647)	**1653**	vw	Vermiculite (1647)	**1653**	vw	Vermiculite (1647)
**1626**	ms	Calcite (1625) / Hematite (1625) / Gypsum (1620)	**1616**	ms	Gypsum (1620)	**1628**	ms	Calcite (1625) / Hematite (1625)
**1587**	vw	Calcite (1588)						
**1560**	vw	Hematite (1562)	**1560**	vw	Hematite (1562)	**1560**	vw	Hematite (1562)
			**1522**	vw	Organics (C=C stretching in conjugated systems)?	**1522**	vw	Organics (C=C stretching in conjugated systems)?
**1508**	vw	Organics (C=C stretching in conjugated systems)?	**1508**	vw	Organics (C=C stretching in conjugated systems)?	**1508**	vw	Organics (C=C stretching in conjugated systems)?
**1489**	vw	Organics (CH bending)?	**1489**	vw	Organics (CH bending)?	**1489**	vw	Organics (CH bending)?
**1474**	vw	Organics (CH bending)?	**1474**	vw	Organics (CH bending)?	**1474**	vw	Organics (CH bending)?
**1456**	vw	Organics (CH bending)?	**1456**	vw	Organics (CH bending)?	**1456**	vw	Organics (CH bending)?
**1437**	vw	Organics (CH bending)?	**1437**	vw	Organics (CH bending)?	**1437**	vw	Organics (CH bending)?
**1420**	vw	Organics (CH bending)?	**1420**	vw	Organics (CH bending)?	**1420**	vw	Organics (CH bending)?
**1350**	s broad	Convolution of many peaks	**1350**	s broad	Convolution of many peaks	**1350**	s broad	Convolution of many peaks
**1339**	w in the broad peak	Organics (C-O stretching)?	**1339**	w in the broad peak	Organics (C-O stretching)?	**1339**	w in the broad peak	Organics (C-O stretching)?
**1315**	w in the broad peak	Organics (C-O stretching)?	**1315**	w in the broad peak	Organics (C-O stretching)?	**1315**	w in the broad peak	Organics (C-O stretching)?
**1281**	m broad	Albite (1277)	**1281**	m broad	Albite (1277)	**1281**	m broad	Albite (1277)
**1159**	w	Quartz (1157 s) / Hematite (1157)	**1159**	m	Quartz (1157 s) / Hematite (1157)	**1159**	m	Quartz (1157 s) / Hematite (1157)
**1120**	s broad	Halite (1109) + Possible convolution of Albite, Quartz, Calcite, Vermiculite Peaks	**1120**	s broad	Possible convolution of Albite, Quartz, Calcite, Vermiculite Peaks	**1109**	s broad	Halite (1109) + Possible convolution of Albite, Quartz, Calcite, Vermiculite Peaks
**1060**	m	Calcite (1065)	**1057**	ms	Calcite (1065)	**1060**	m	Calcite (1065)
**1022**	w	Hematite (1022)	**1022**	w	Hematite (1022)	**1022**	w	Hematite (1022)
**945**	m broad	Calcite (945)	**935**	m broad	Calcite (935)?	**935**	m broad	Calcite (935)?
**870**	w	Calcite (872)	**870**	w	Calcite (872)	**870**	w	Calcite (872)
**847**	w	Calcite (847)	**847**	w	Calcite (847)	**847**	w	Calcite (847)
**715**	very broad	Convolution of many peaks with Calcite (712) the most important	**720**	very broad	Convolution of many peaks	**720**	very broad	Convolution of many peaks
**638**	n the broad p	Albite (633)	**635**	n the broad p	Albite (633)	**638**	n the broad p	Albite (633)
**613**	vw	Halite (619)	**613**	vw	Halite (619)	**613**	vw	Halite (619)
**567**	m broad	Quartz (571)	**571**	m broad	Quartz (571)	**561**	m broad	Albite (555) / Quartz (571)
**515**	m	Quartz (522)	**520**	m	Quartz (522)	**515**	m	Quartz (522)
**455**	m	Albite (455)	**455**	m	Albite (455)	**455**	m	Albite (455)
			**440**	vw	Calcite (436)			
			**432**	vw	Analcime (434)	**428**	vw	Analcime (434)
**426**	vw	Albite (420)						
**403**	w	Quartz (403)	**403**	m	Quartz (403)	**405**	m	Quartz (403)

Supplementary Figures

Supplementary Figures S1 to S18

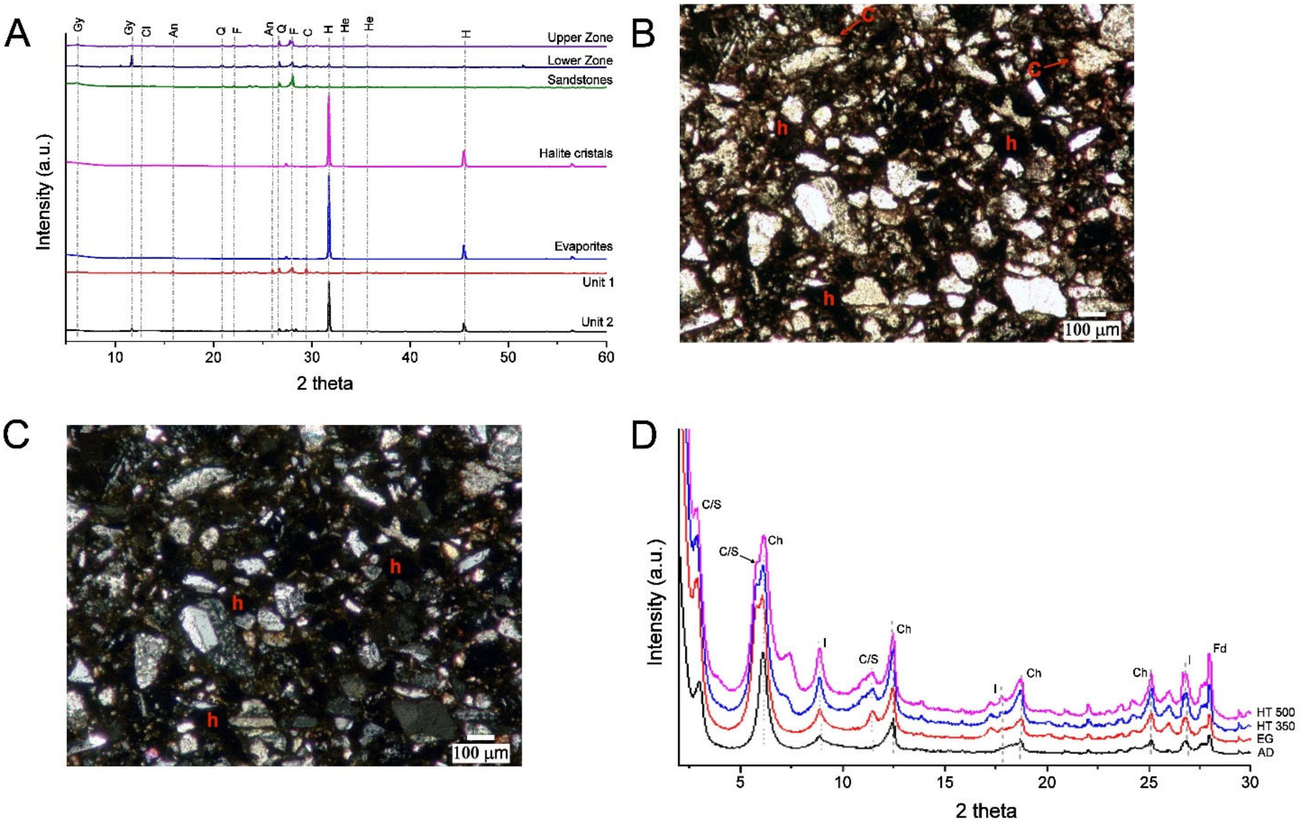

Fig. S1. Mineralogic and petrographic characterization of Red Stone samples. A, X-Ray diffraction (XRD) patterns of Red Stone samples. Quartz (Q), Albite (Al), Calcite (C), Halite (H), Hematite (He), Analcime (An), Gypsum (Gy), Vermiculite (V), Clinochlore (CI). Thin sections of sample A06 with (B) no polarized, and (C) polarized light. Hematite crystals (h) are present in the fine-grain sandstone as a supporting matrix (dominated by subangular crystals of quartz, plagioclase and rock fragments), as wells as pellet coatings (c). D, X-Ray diffraction (XRD) patterns of AD (air dried), EG (ethylene glycol solvated, HT 350 (heated at 350°C) and HT 500 (heated at 500°C) mudstone samples. Chlorite/Smectite (C/S), Chlorite (Ch), illite (I), feldspar (Fd).

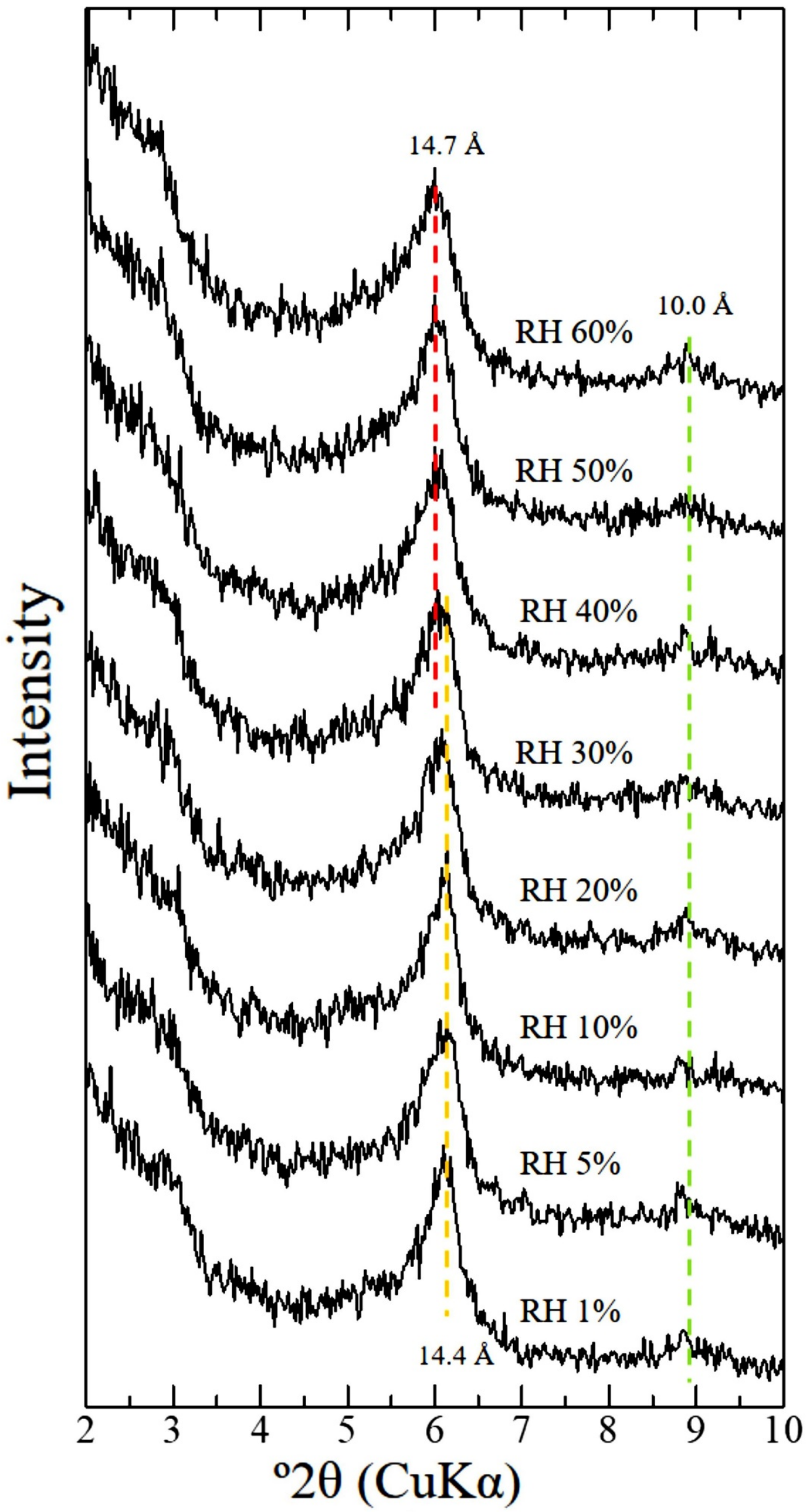

Fig. S2. XRD patterns of oriented clay-fraction from mudstone samples under different relative humidity (RH).

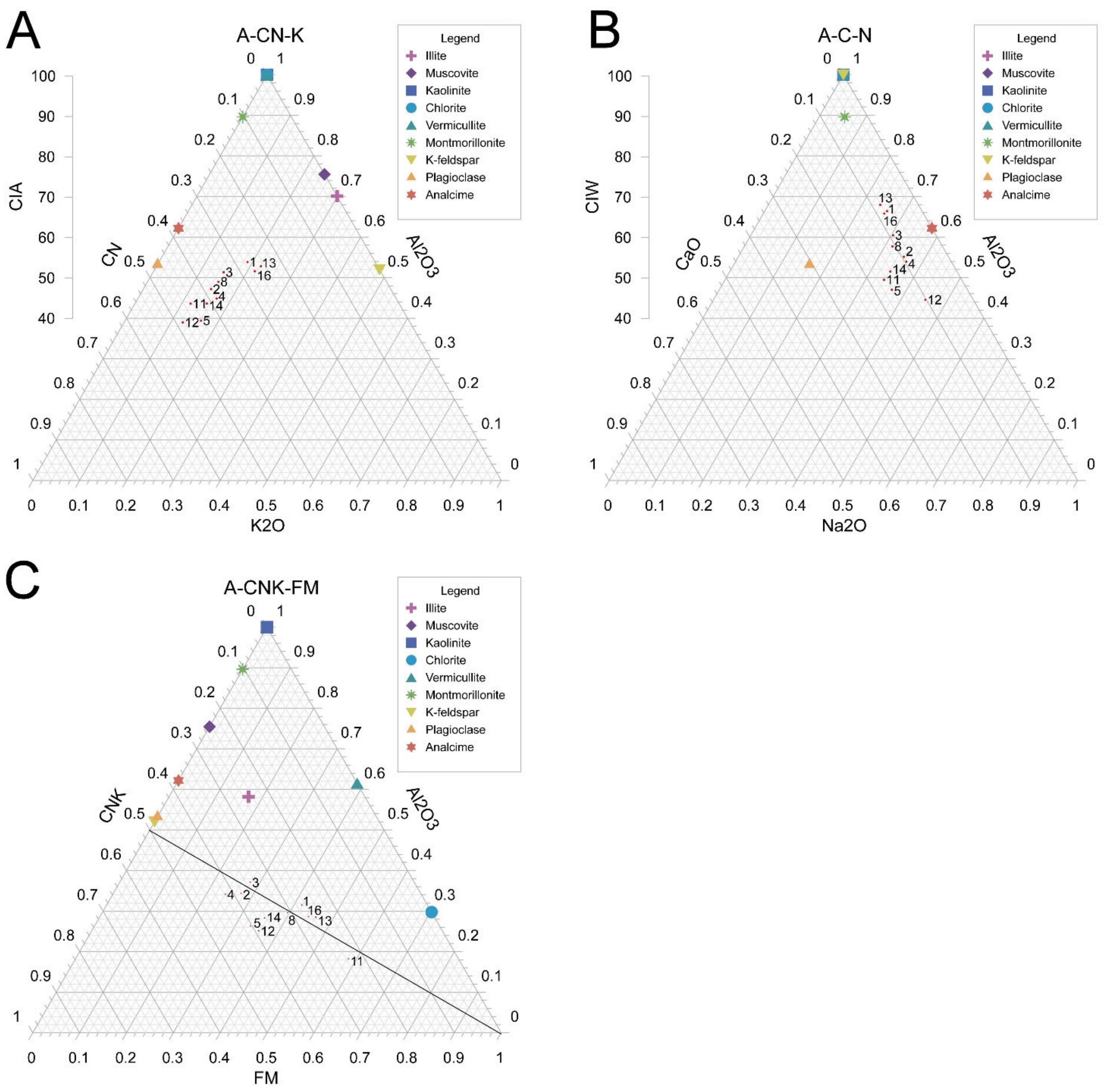

Fig. S3. Geochemical analyses of the silicate fraction of Red Stone samples. (A) A-CN-K ternary diagram. (B) A-C-N ternary diagram. (C), A-CNK-FM ternary diagram. In all cases black numbers show the numbering of A1 to A16 Red Stone samples. Other colors and shapes represent references samples.

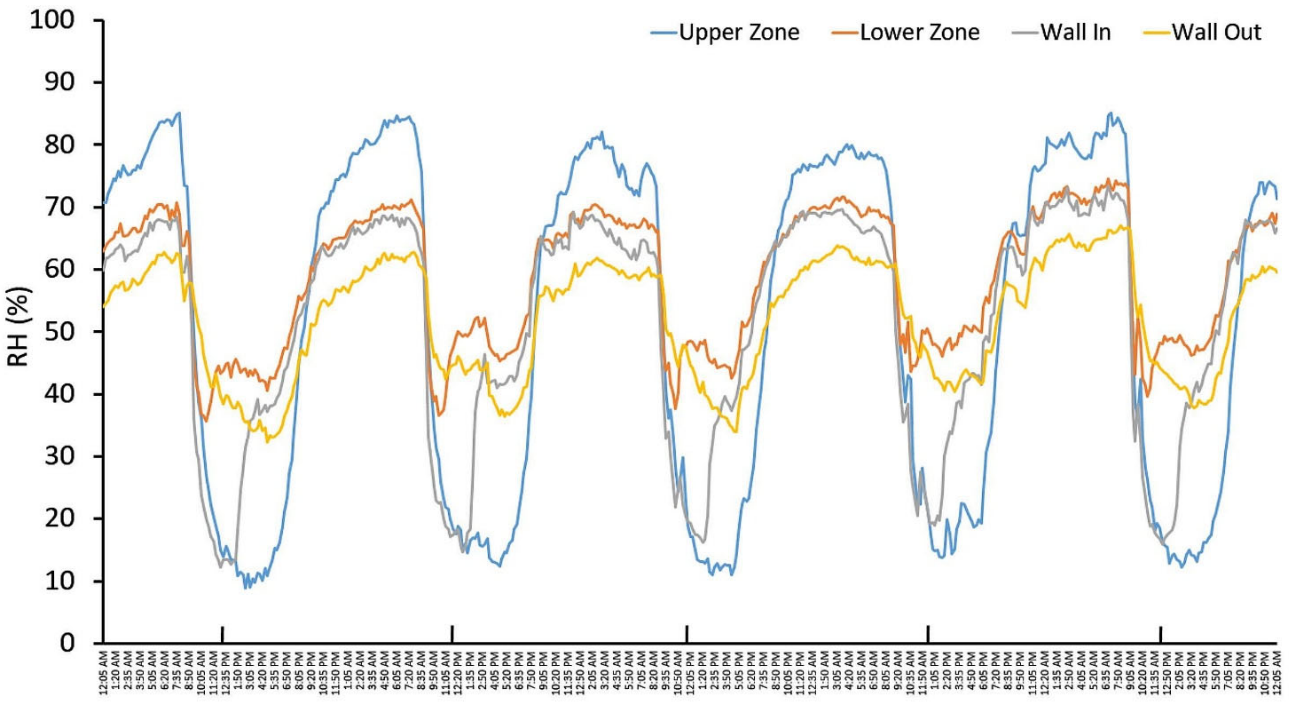

Fig. S4. Temporal behavior of relative humidity (RH) conditions at the Red Stone site. RH changes over the course of five representative days. Larger marks in the x axis show noon time of each day. Wall in and wall out refer to the sensors pointed to the inside volume of the inspected gully or away from it.

A

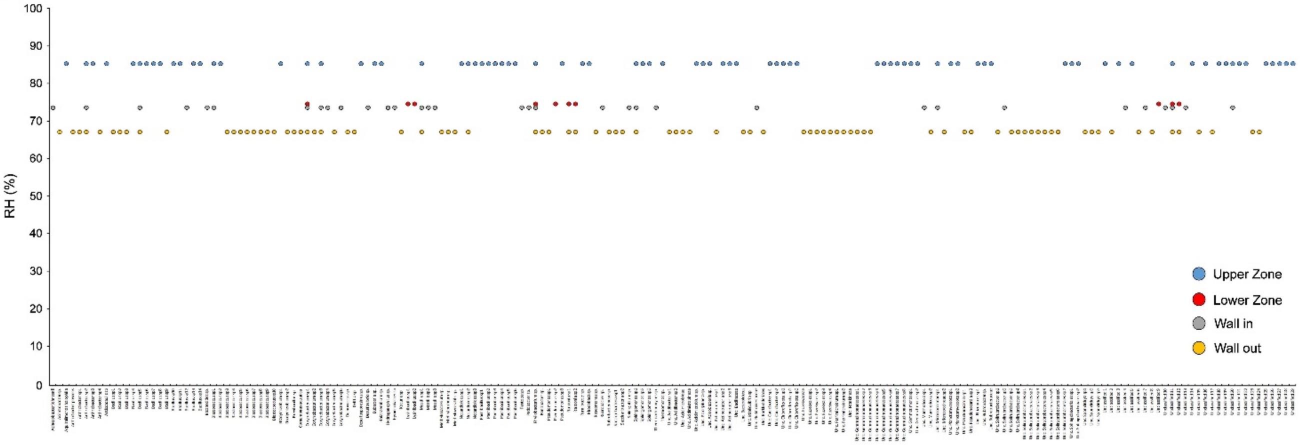

B

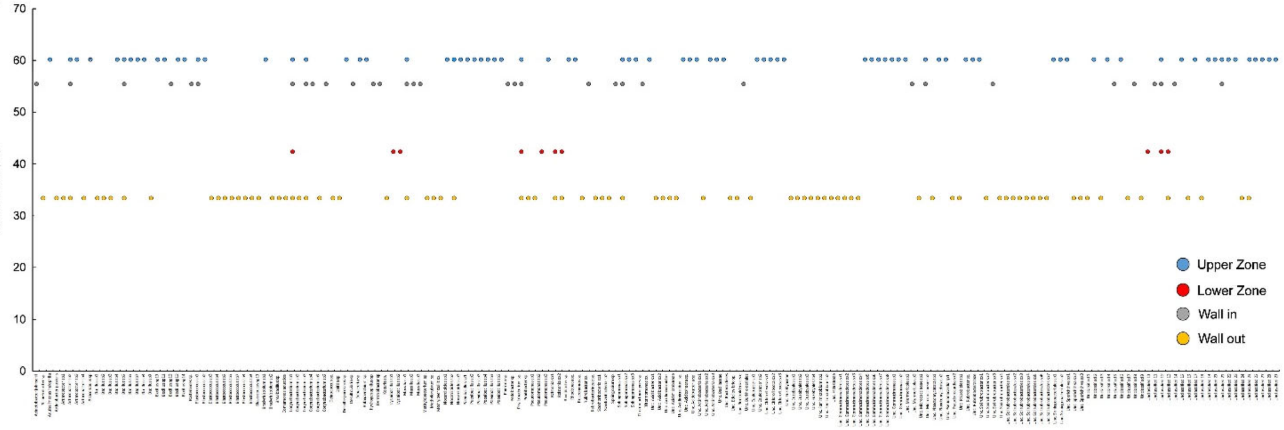

Fig. S5. Next-Generation Sequencing (NGS) diversity correlations with environmental factors

(A) NGS diversity correlation with maximum relative humidity values. (B) NGS diversity correlation with maximum temperature values. Sensor location can be seen in Figure 1, and detailed NGS biodiversity in Table S1.

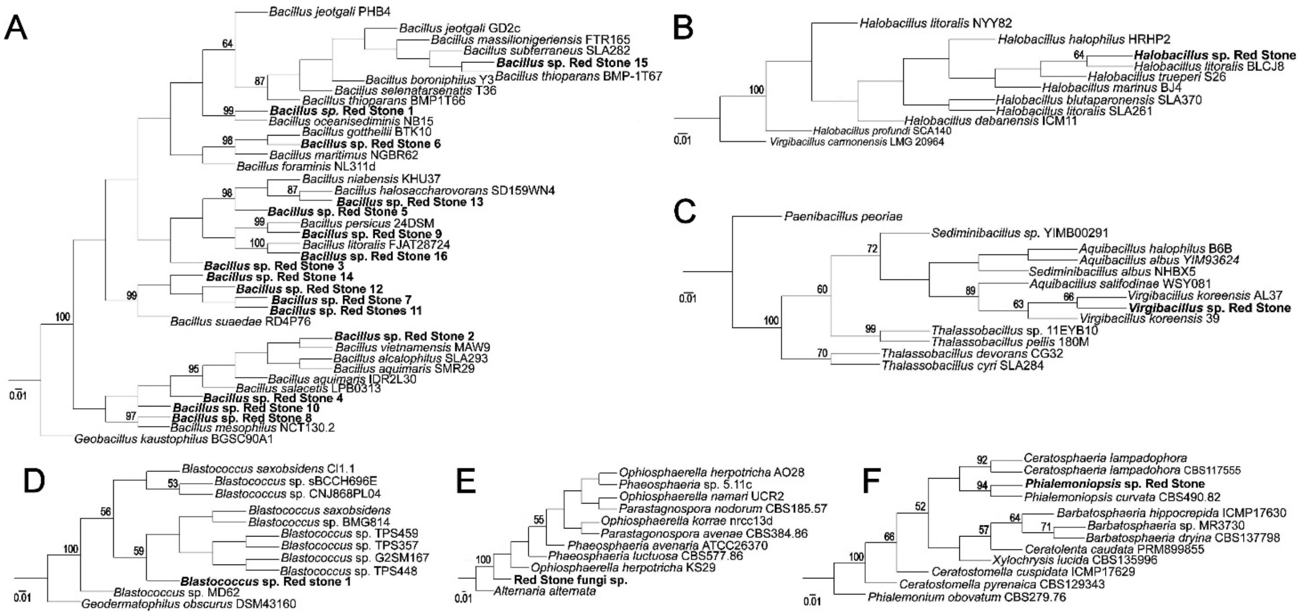

Fig. S6. Closest phylogenetic matches of microbial isolates found in Red Stone Samples.

Neighbour joining phylogenetic trees based on 16S rRNA and 18S rRNA gene sequences of Red Stone isolates and their closely related species from genus (A) *Bacillus*, (B) *Halobacillus*, (C) *Virgibacillus*, (D) *Blastococcus*, (E) a yet unidentified fungi and (F) *Phialemoniopsis*. Number at the nodes indicate bootstrap values based on 10000 resampled datasets.

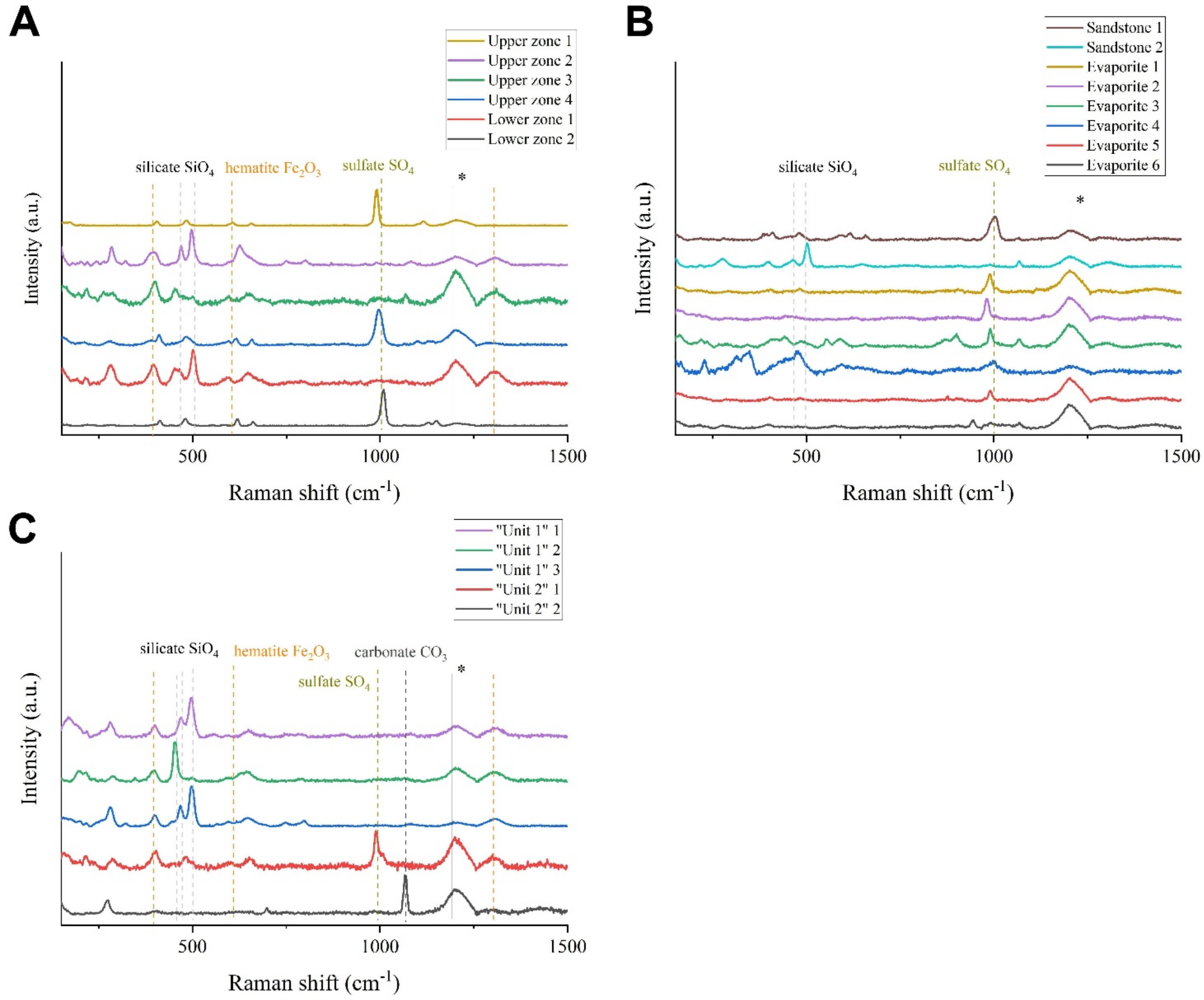

Fig S7. Raman spectra of Red Stone samples (Minerals). (A) Raman spectra of Upper Zone and Lower Zone samples. (B) Raman spectra of Sandstones and Evaporite samples. (C) Raman spectra of Unit 1 and Unit 2 samples. Raman spectra were taken in different spots of the samples analyzed, where hematite, quartz and albite have been identified by comparison with the ADAMM database. Raman spectra of organics are shown in panels C-F of figure 3.

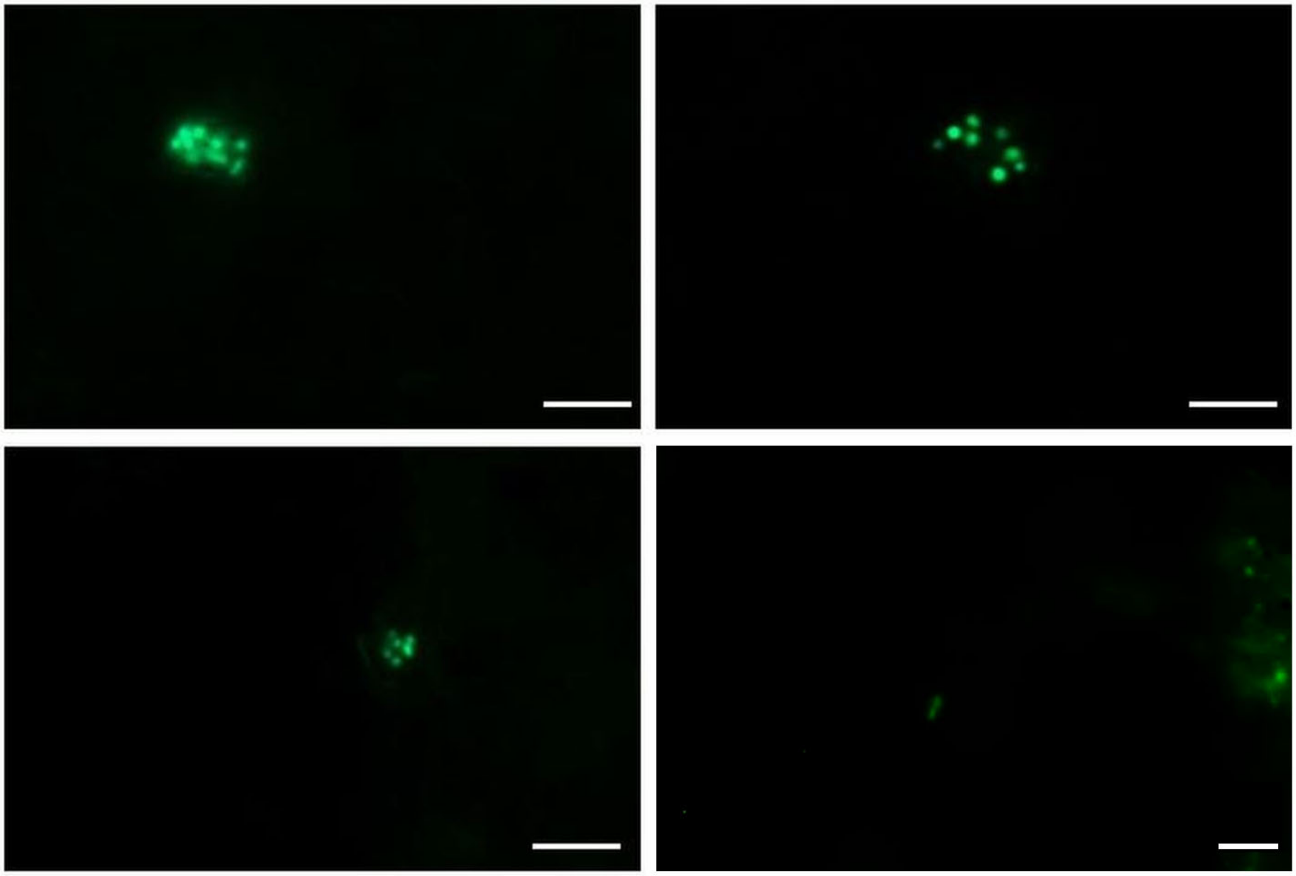

Fig S8. SYBR Green DNA staining of a lower zone sample.

Fluorescence microscopy images of samples stained with SYBR Green. Bars in all cases represent 5 µm.

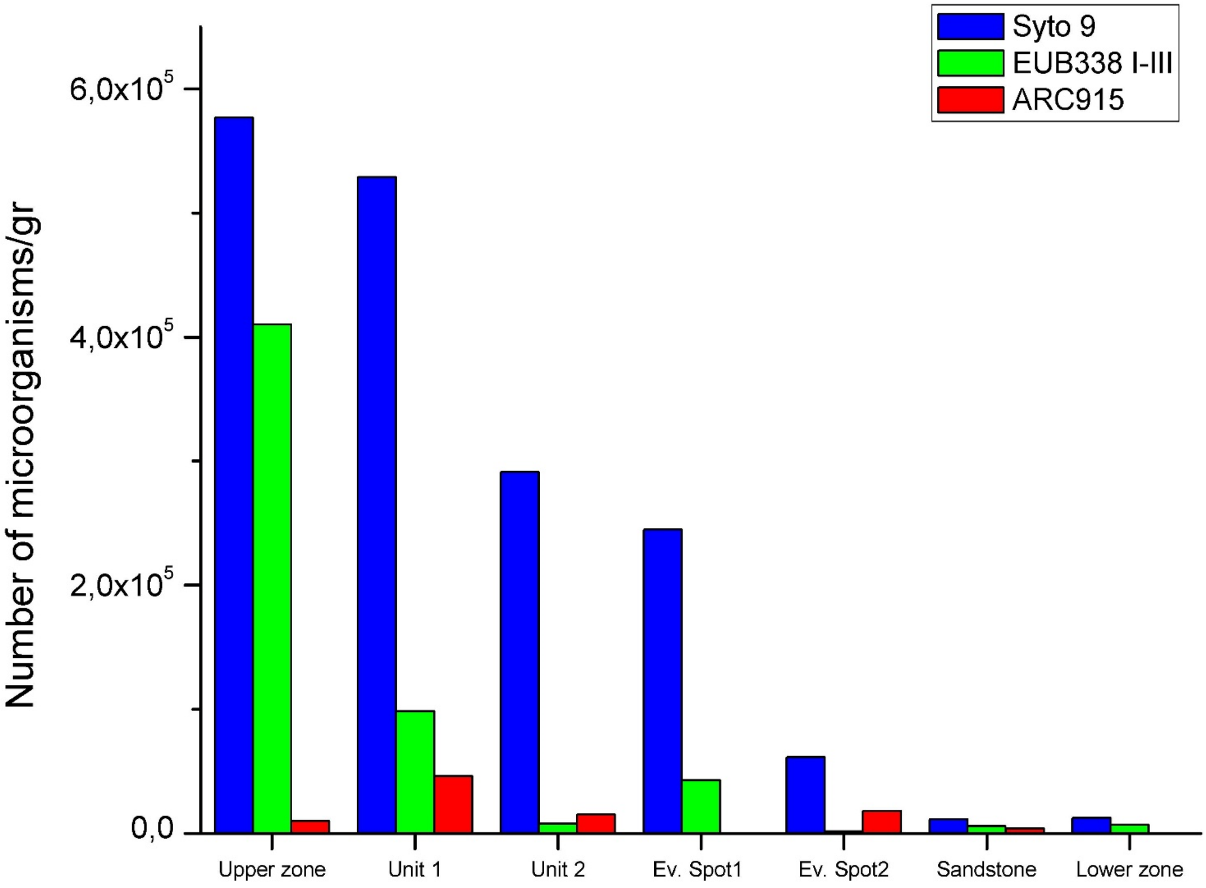

Fig. S9. Catalyzed reporter deposition fluorescence in situ hybridization (CARD-FISH) analyses of Red Stone Samples. In blue, total number of microorganisms detected by general DNA staining with Syto9. In green, number of bacteria detected by CARD-FISH with EUB 338 I-III probes. In red, number of archaea detected by CARD-FISH with the ARC915 probe.

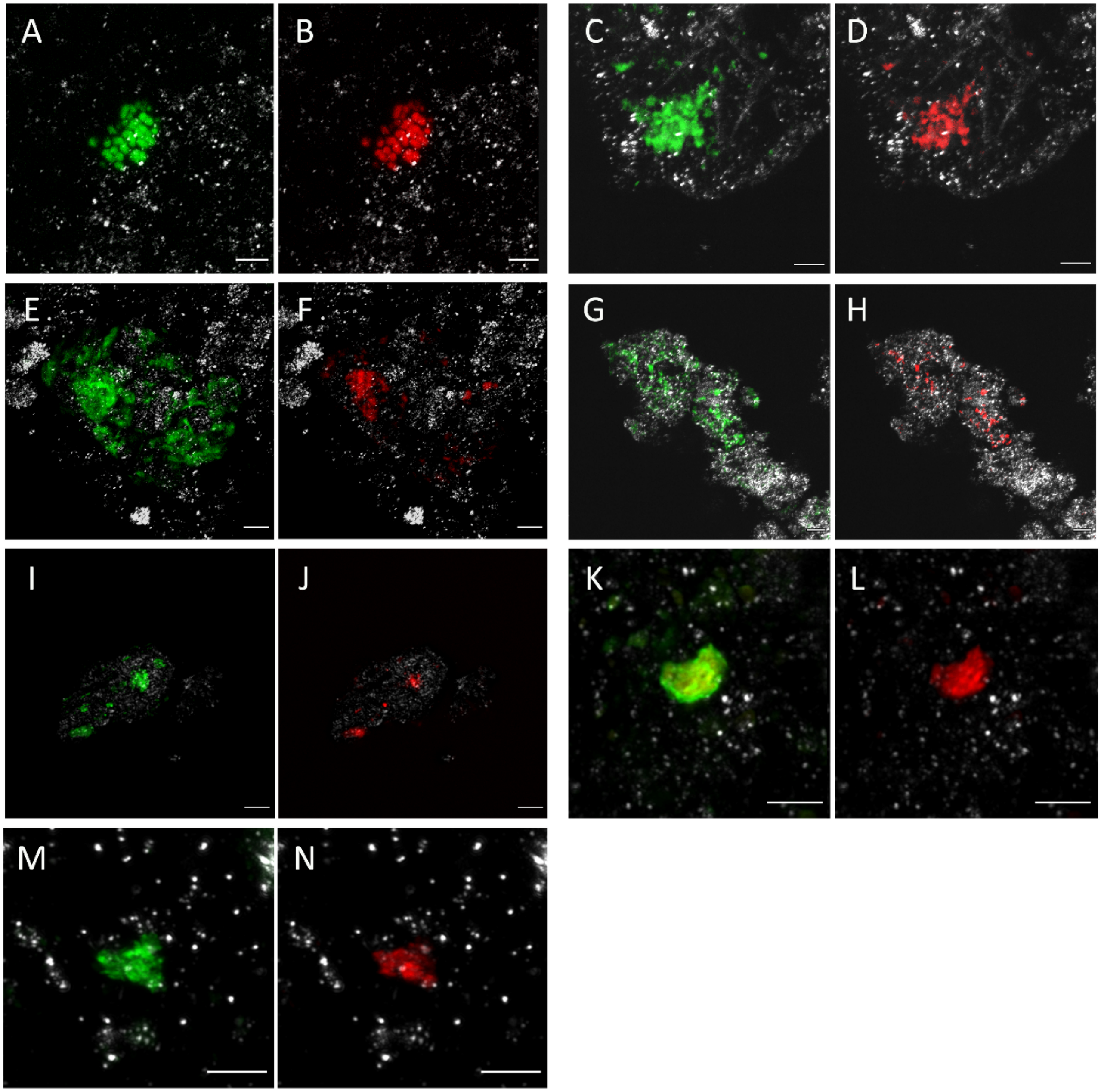

Fig. S10. Bacteria and Archaea detected by CARD-FISH using EUB 338 I-III and ARC195 probes in the Red Stone samples. A-B) Bacteria, Upper zone. C-D) Bacteria, Unit 1. E-F) Archaea, Unit 2. G-H) Bacteria, Evaporites, Spot 1. I-J) Archaea, Evaporites Spot 2. K-L) Bacteria, sandstones. M-N) Bacteria, Lower zone. In green, DNA general stain. In red, CARD-FISH probe signal. In grey, reflection. Scale bars, 5µm.

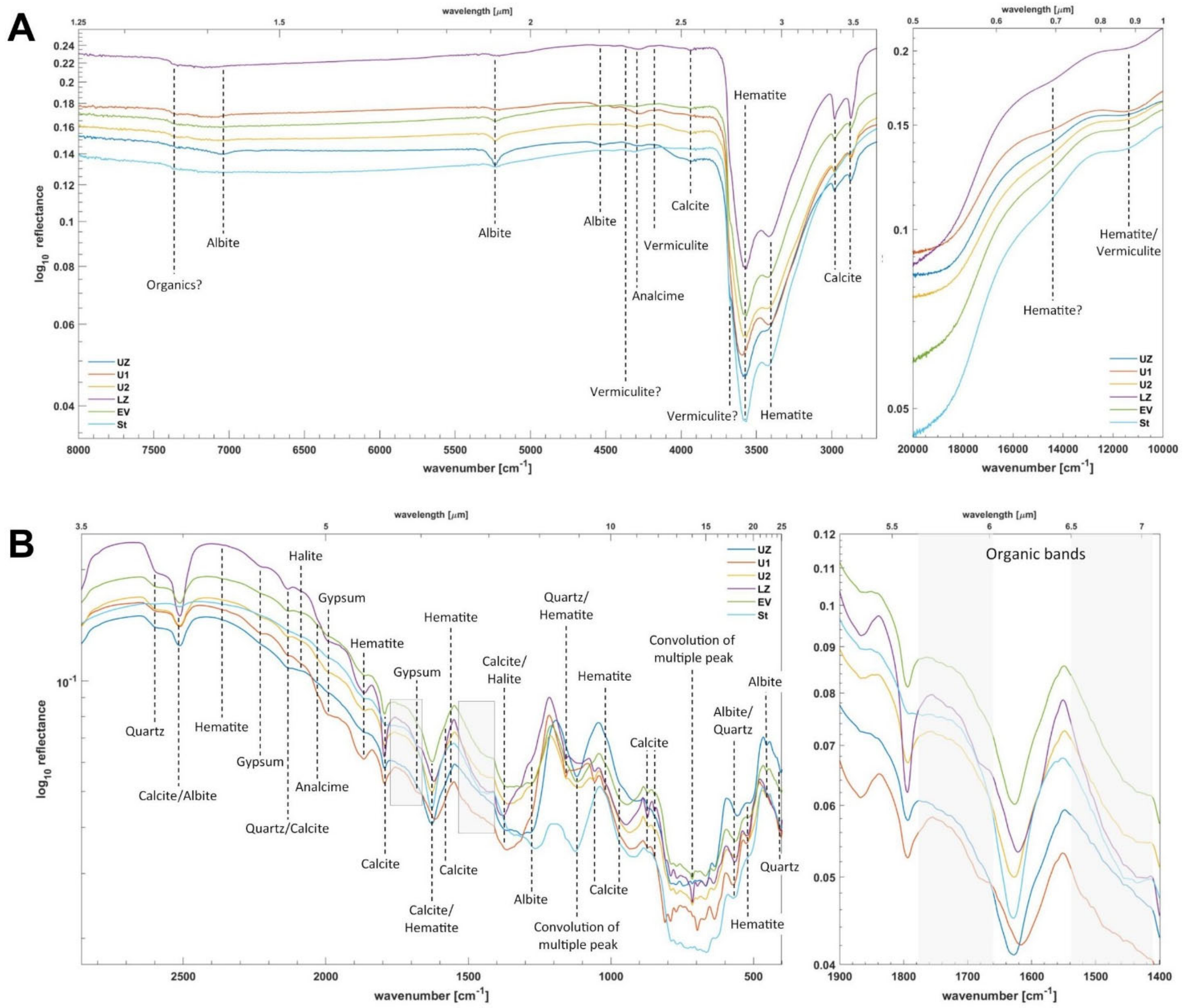

Fig. S11. Diffuse reflectance infrared fourier transform spectroscopy (DRIFTS) spectra of Red Stone samples. A, NIR spectral region, along with peak assignments. B, MIR spectral region, along with peak assignments. Bands of organics have been highlighted in gray in the inset.

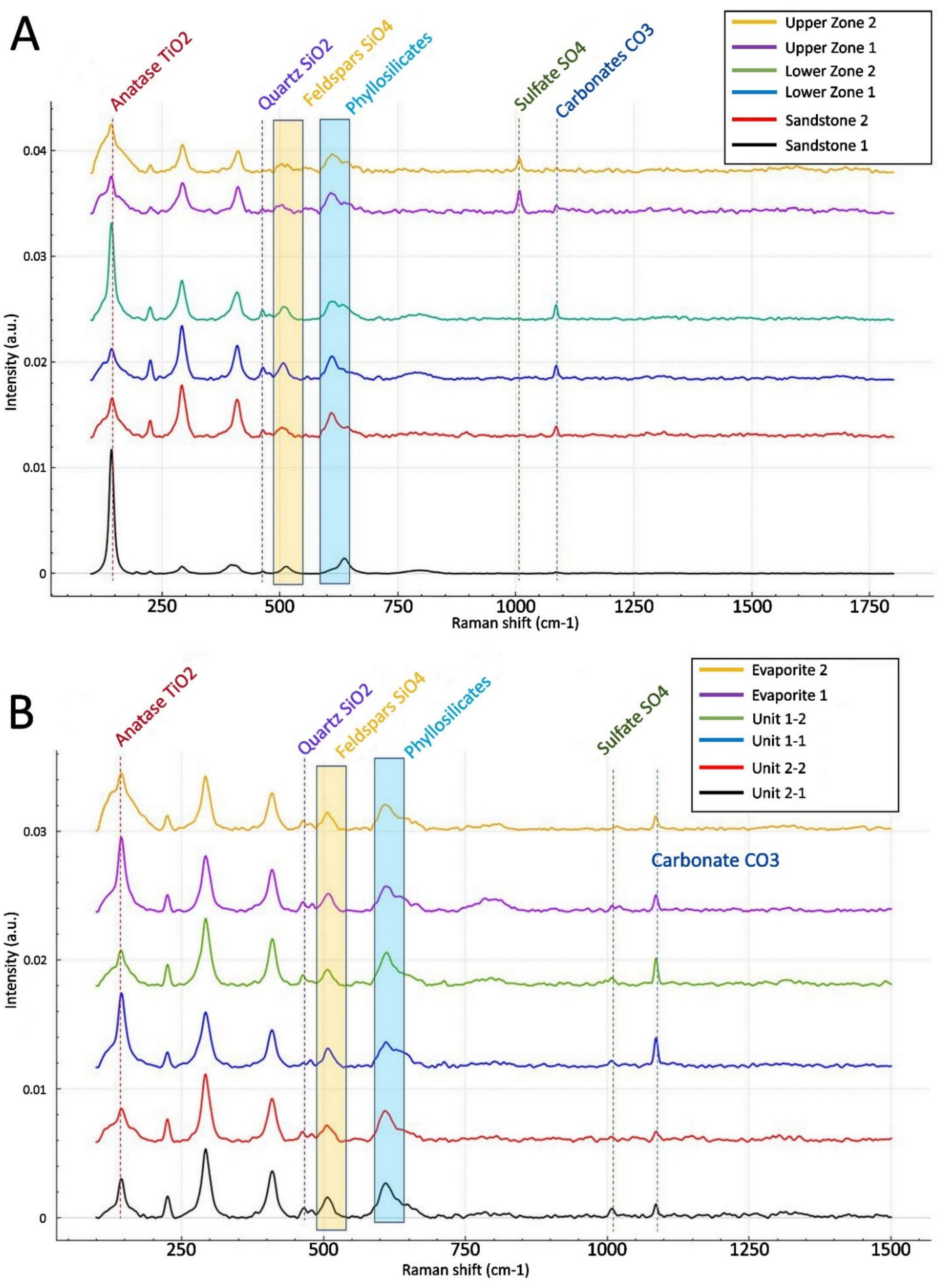

Fig. S12. Fourier Transform (FT) Raman spectra of Red Stone samples. (A) FT-Raman spectra of Upper Zone, Lower Zone and Sandstones samples. (B) Raman spectra of Unit 1, Unit 2 and Evaporite samples. Identification was done by comparison with the ADAMM database.

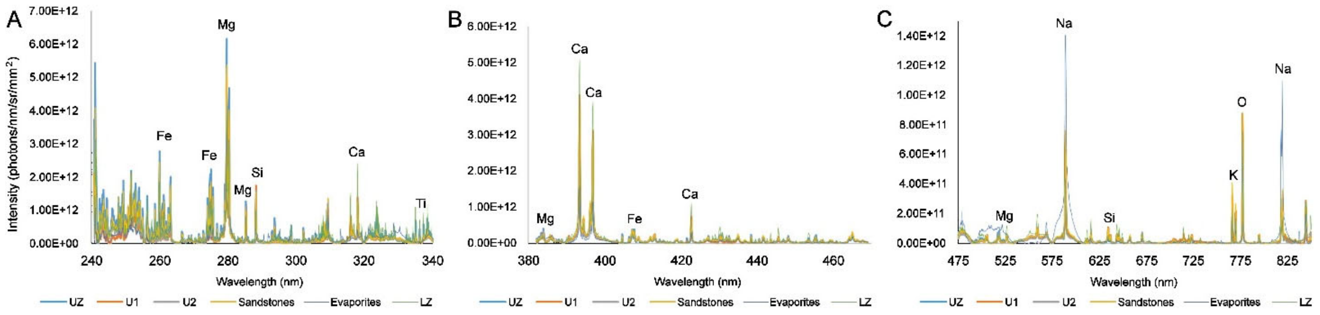

Figure S13.- Laser Induced Breakdown Spectroscopy (LIBS) spectra of Red Stone samples. Panels show the three spectral ranges covered by the instrument A, UV; B, Violet-Blue; C, VNIR. Major emission lines are labeled.

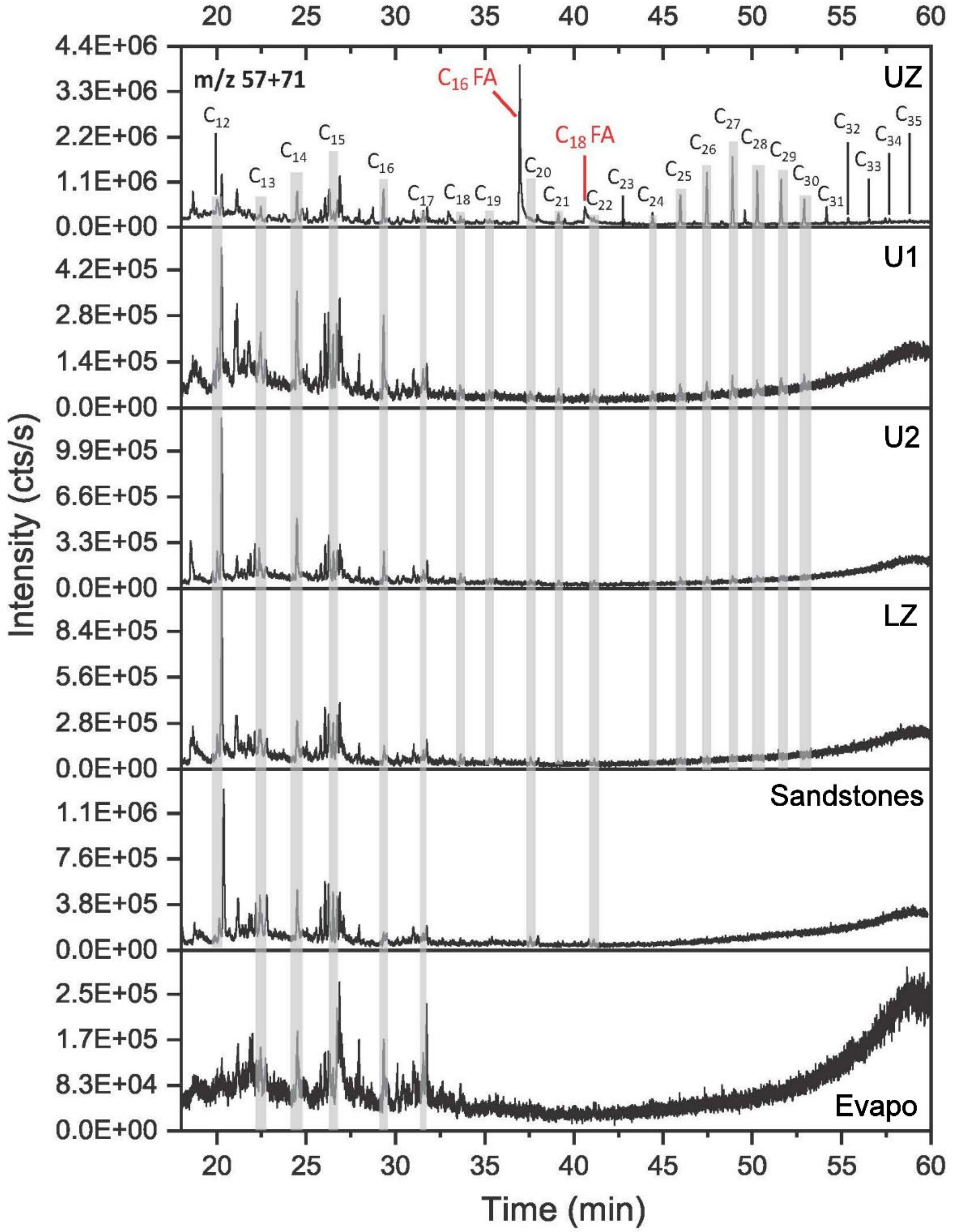

Fig S14. Reconstructed ion chromatogram obtained after the SAM-like pyrolysis of the Red Stone samples and plotted as: m/z 57 + m/z 71. The SAM-like pyrolysis corresponds to an initial temperature of 40°C up to 850°C at a 35°C/min rate. The compounds highlighted in grey correspond to the C12-C35 alkanes detected in the Upper Zone (Uz) sample. C12-C30 were detected in Upper Unit 1 and 2 (U1 and U2), and in the Lower Zone sample (Lz), C12-C17 in addition to C20 and C22 were detected in the sandstone sample and only C13-C17 in the evaporite sample (EVAP).

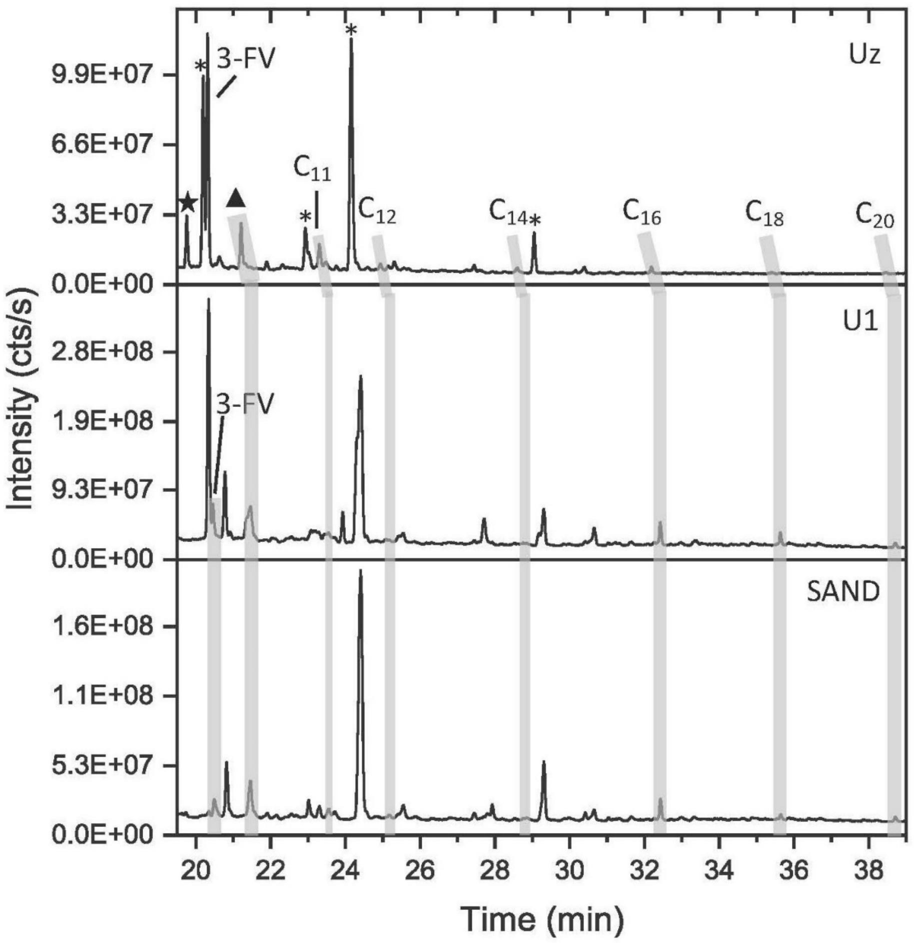

Fig. S15. Total Ion Chromatograms (TIC) obtained after the SAM-like derivatization of the Upper Zone, Unit 1, and Sandstone samples and highlighting the detection of C11, C12, C14, C16, C18 and C20 fatty acids. The star symbol points to proline and the triangle to the unsaturated carboxylic acid butenoic acid. 3-FV: 3-fluorovaline amino acid internal standard. *MTBSTFA byproducts.

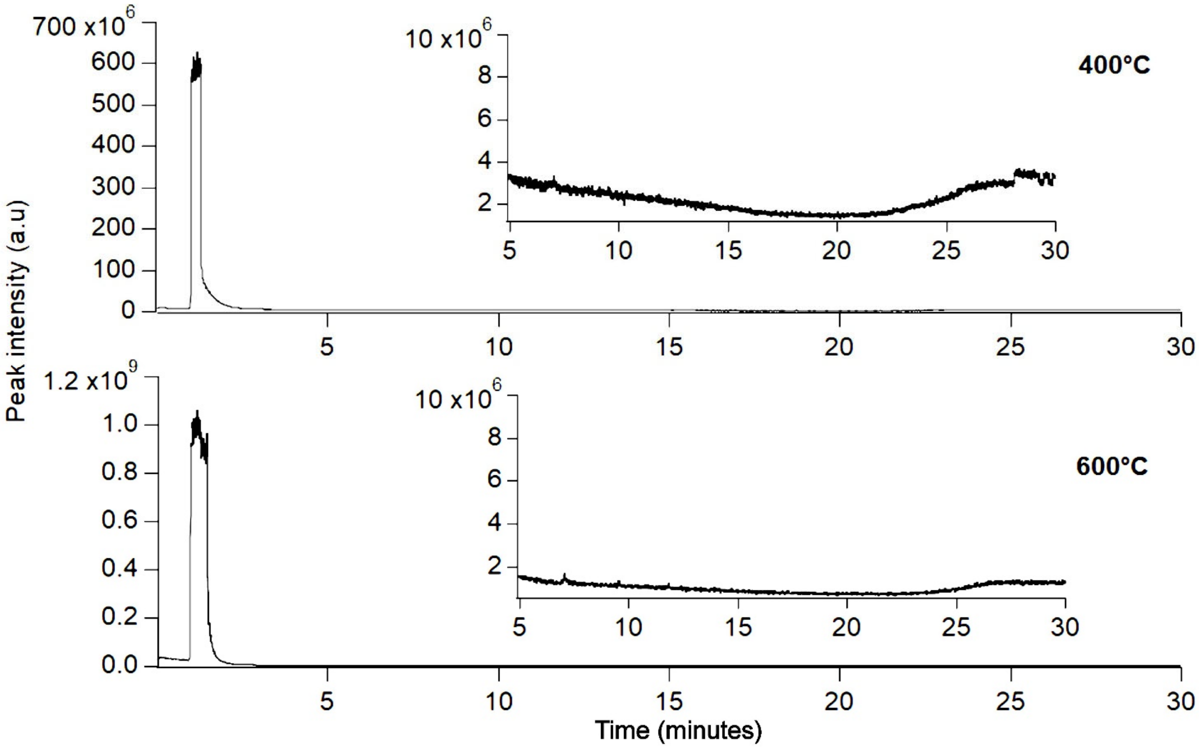

Fig. S16. Chromatograms obtained after flash pyrolysis at 400°C and 600°C of red stone samples. As no detection was possible, only the sandstone sample is show as a representative. The wide peak at minute 1 is evolved water from the sample.

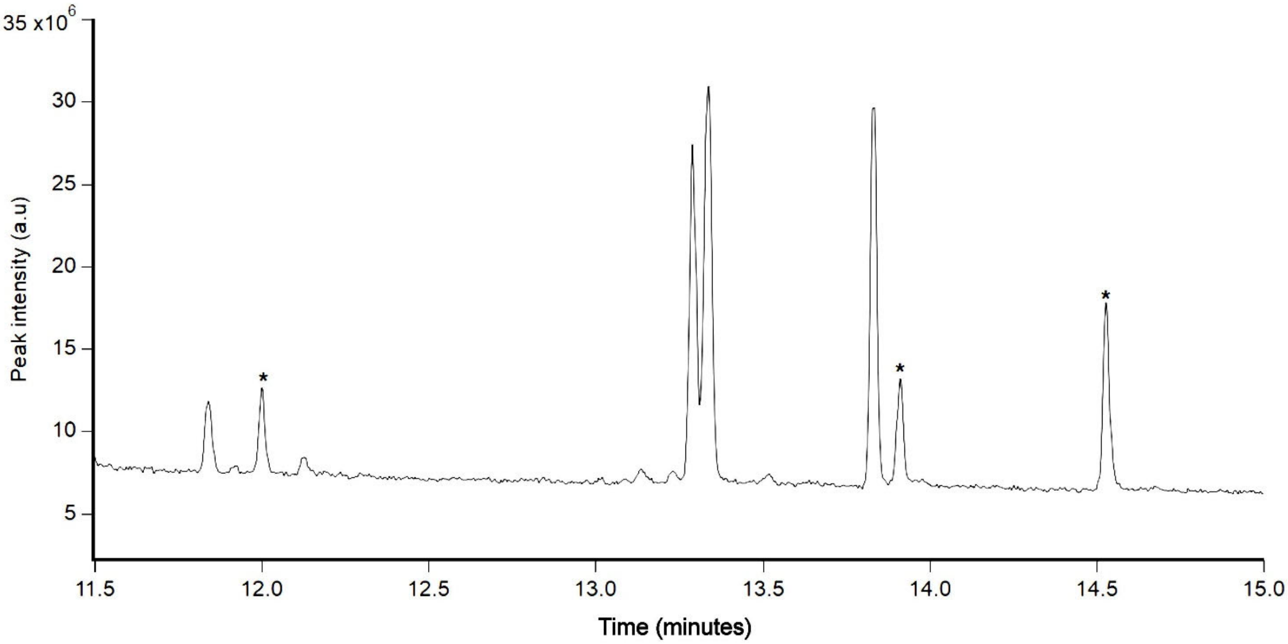

Fig. S17. Portion of the chromatogram obtained after direct derivatization with MTBSTFA-DMF reagent of the evaporites sample. The peaks marked with asterisks corresponds to derivatized organic compounds (attributed to aliphatic carboxylic acids but no strict identification could be found). The other peaks are either byproducts of the derivatization process or column artifacts.

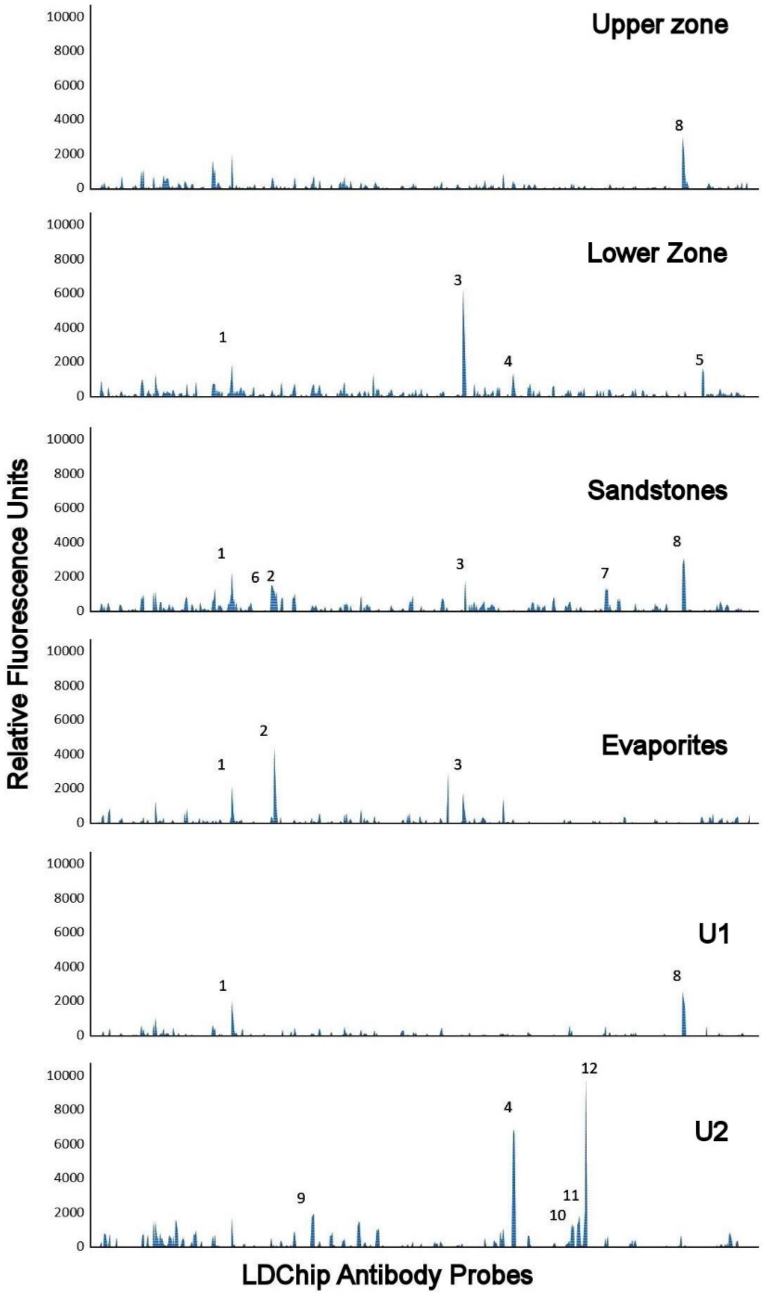

Fig. S18. Signs of life detector (SOLID)-LDChip detection of microbial biosignatures in Red Stone samples. Immunograms showing the quantified relative fluorescence obtained from LDChip microarray immunoassays after subtracting blank controls (see methods). Numbers indicate those positive immunodetections with antibodies produced against: 1, *Desulfovibrio vulgaris*; 2, *Streptomyces* spp (Actinobacteria); 3, *Rivularia* sp. (Cyanobacteria); 4, HscA chaperon protein for Fe-S cluster assembly; 5, Water stress and hypersensitive response (WHy) domain containing protein from bacteria (*Pseudomonas*); 6, Spores from *Streptomyces* sp.; 7, Nitrite Oxidoreductase protein from *Nitrobacte*r; 8, Phycocyanin subunit alpha (Cyanobacteria); 9, *Bacillus* sp. (Firmicutes); 10, Nitrogenase NifH peptide; 11, Nitrogenase NifD peptide; 12, Nitrogenase NifS peptide.

Total Ion Chromatograms (TIC)

Raw lipid chromatograms in the acidic (Fig. C1-C8) and apolar (Fig. C9-C17) lipid fractions of the Red Stone. All chromatograms are shown as Total Ion Chromatograms (TIC), except for Fig. C17, where hopanes were identified in the partial ion chromatogram m/z=191. In all chromatograms, the numbers over the peaks indicate the number of carbons; and IS means internal standard (i.e. tetracosane-D_{50} and myristic acid-D_{27} in the apolar and acidic fractions, respectively). In Fig. C17, R and S refer to the right hand and left hand enantiomers of the C_{31} αβ hopanoid, respectively. The abundant unidentified peaks in Fig. C7 and C8-C16 correspond largely to xyloxane compounds from column bleeding.

Fig C1. Upper Zone (fatty acids)

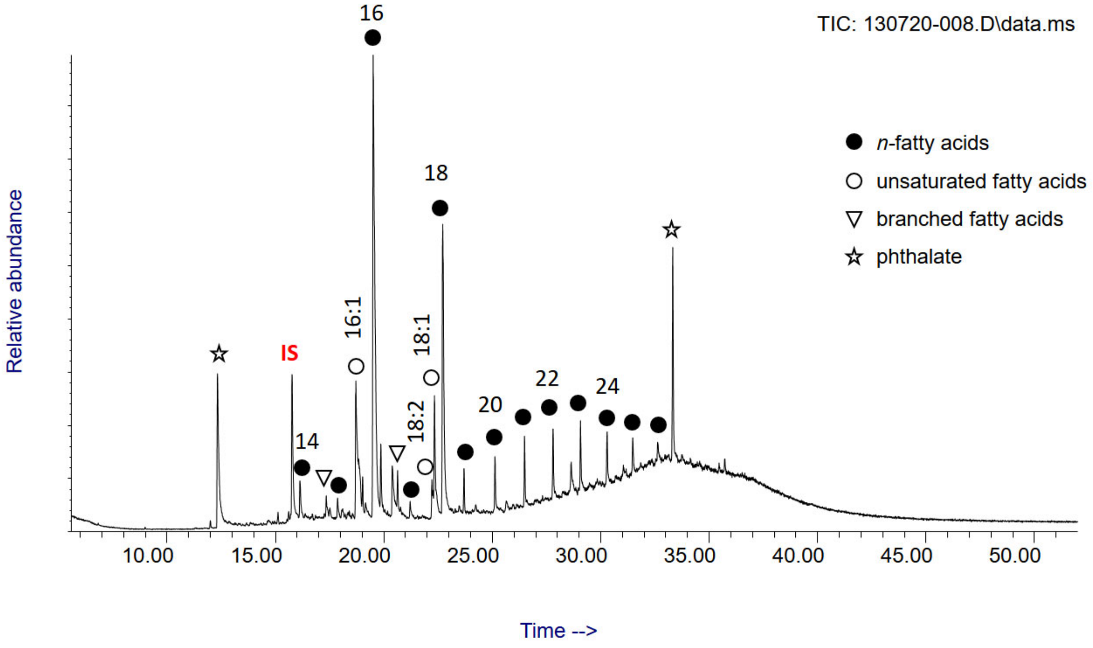

Fig C2. Lower Zone (fatty acids)

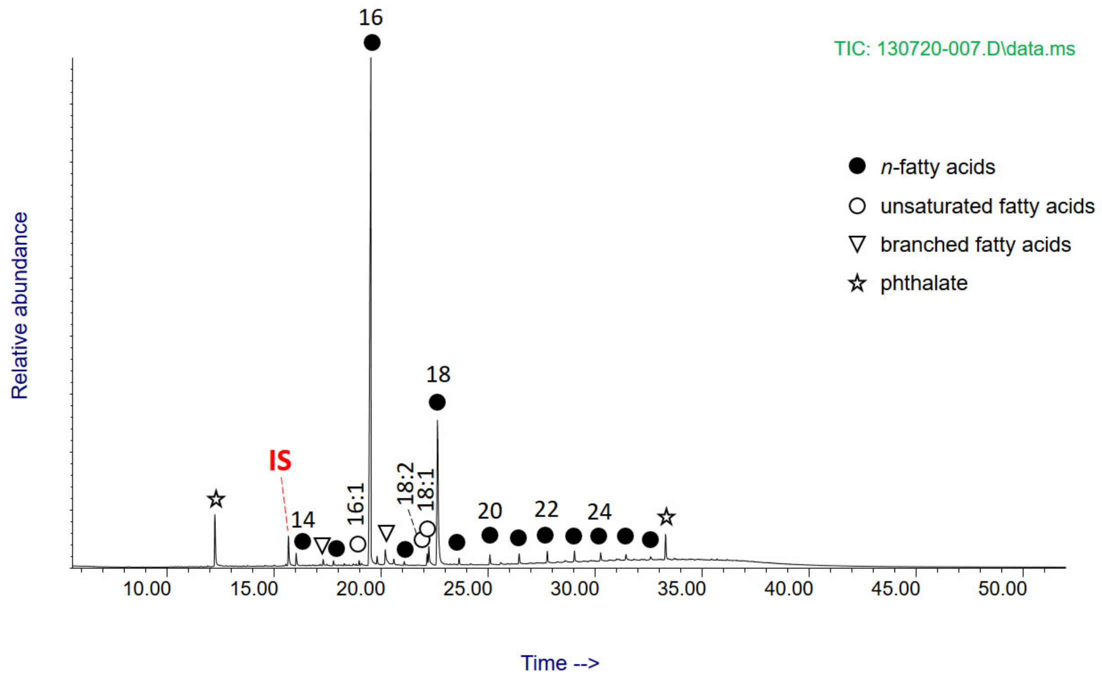

Fig C3. Sandstone (fatty acids)

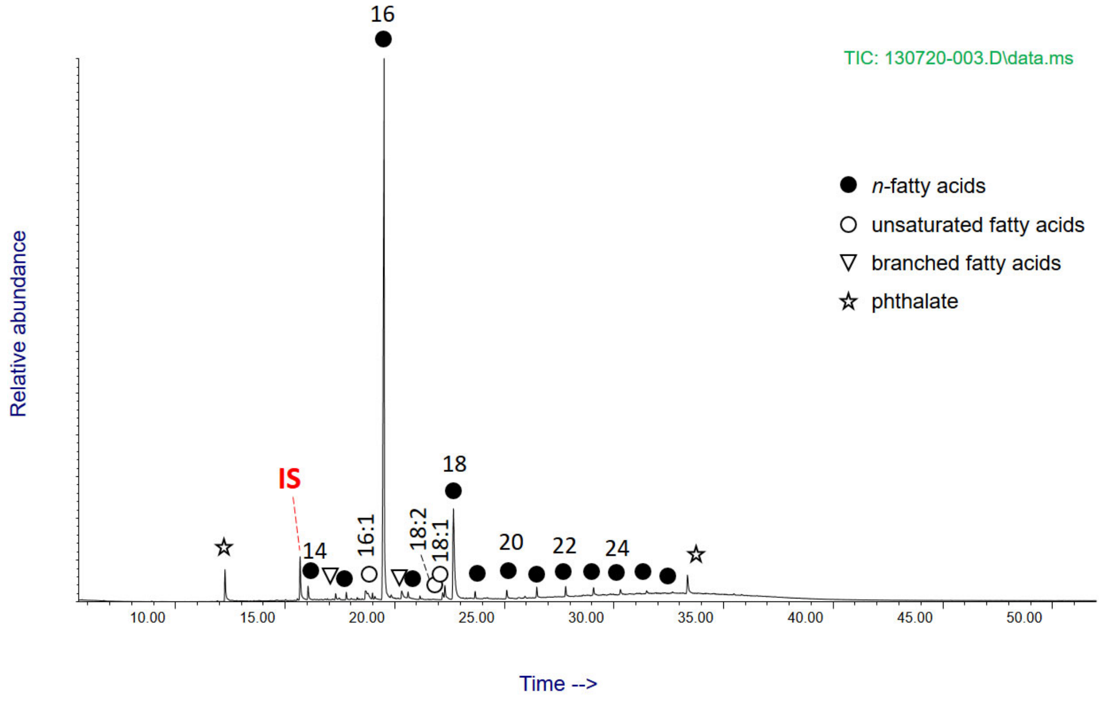

Fig C4. Evaporites spot 1 (fatty acids)

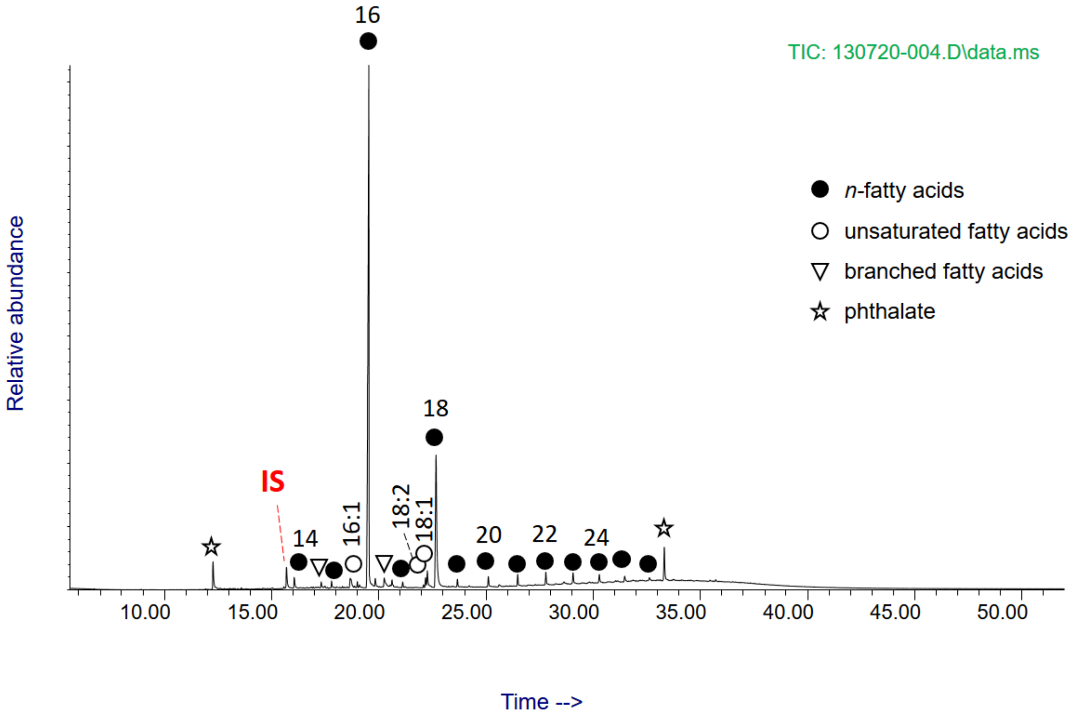

Fig C5. Evaporites spot 2 (fatty acids)

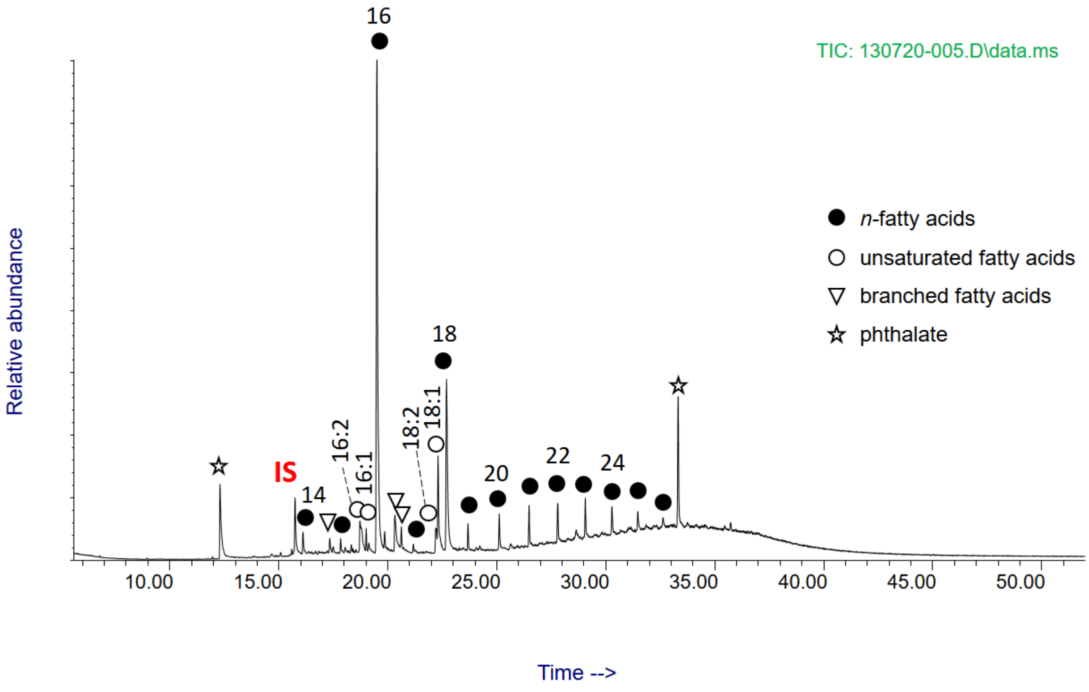

Fig C6. U1 (fatty acids)

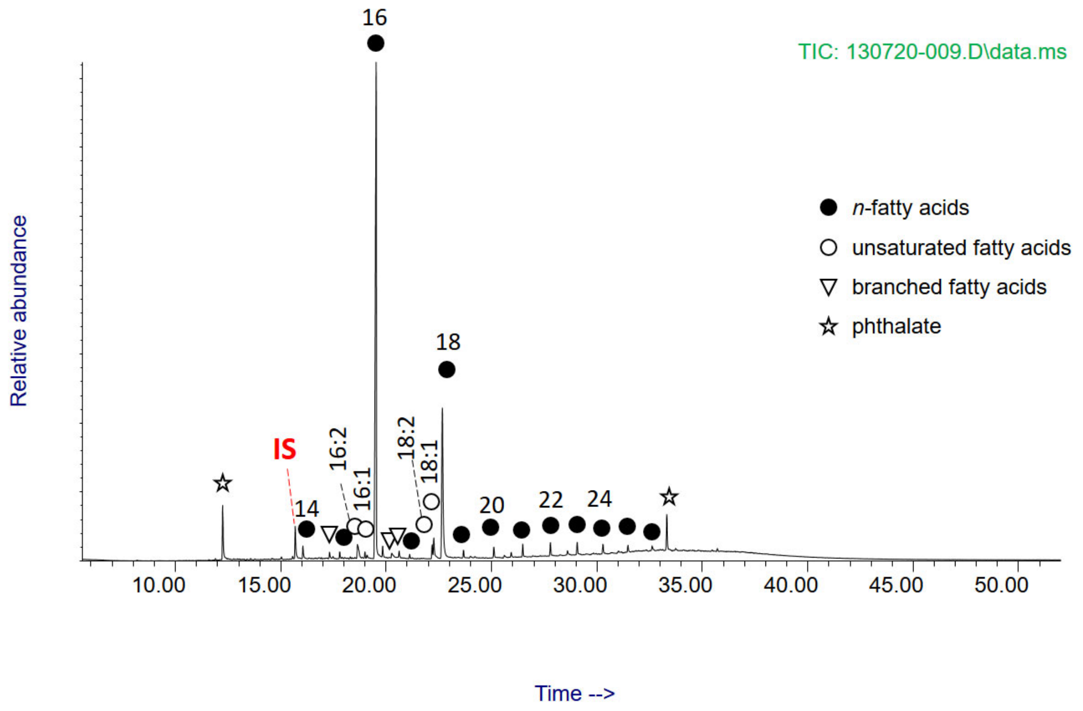

Fig C7. U2 (fatty acids)

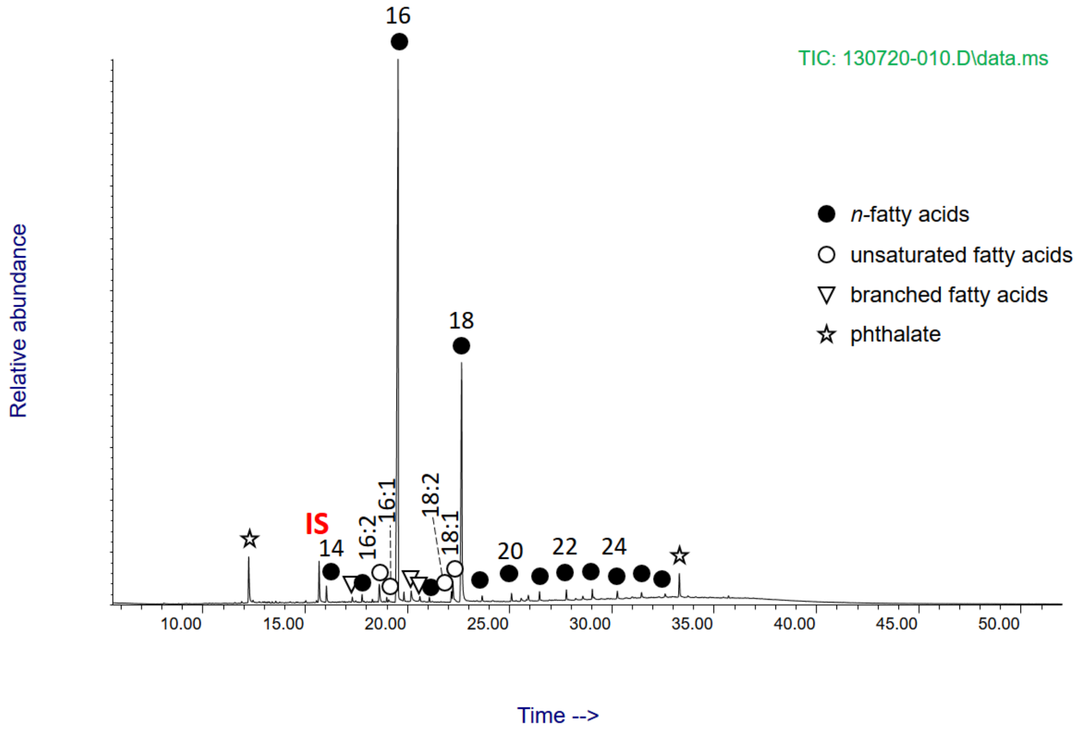

Fig C8. Mudstones (fatty acids)

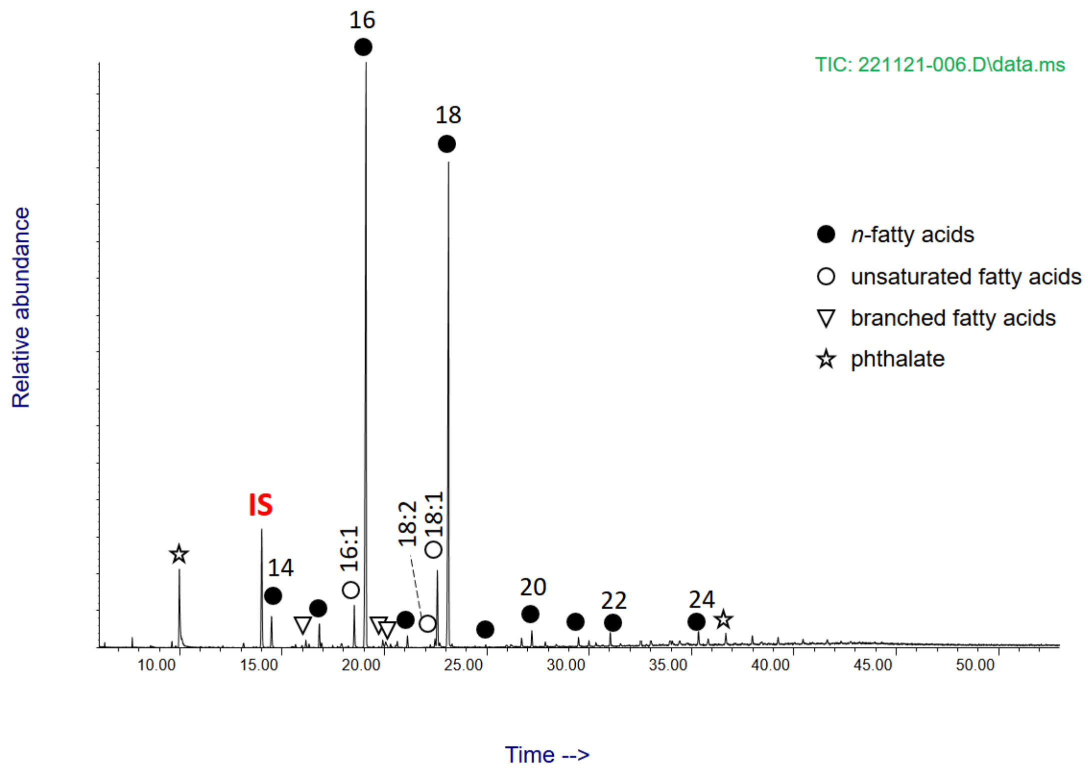

Fig C9. Upper Zone (hydrocarbons)

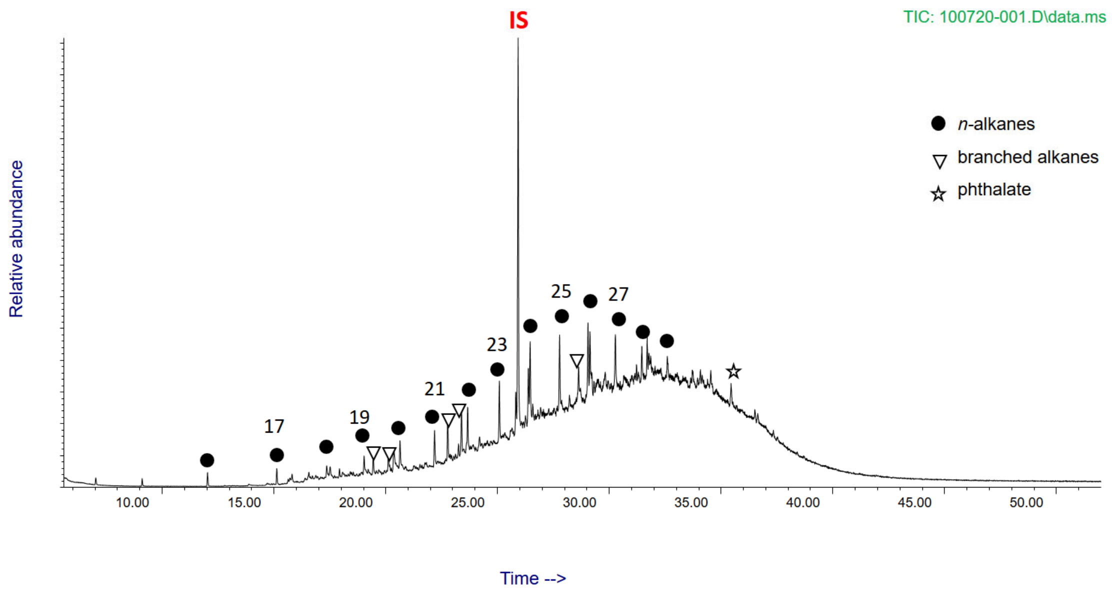

Fig C10. Lower Zone (hydrocarbons)

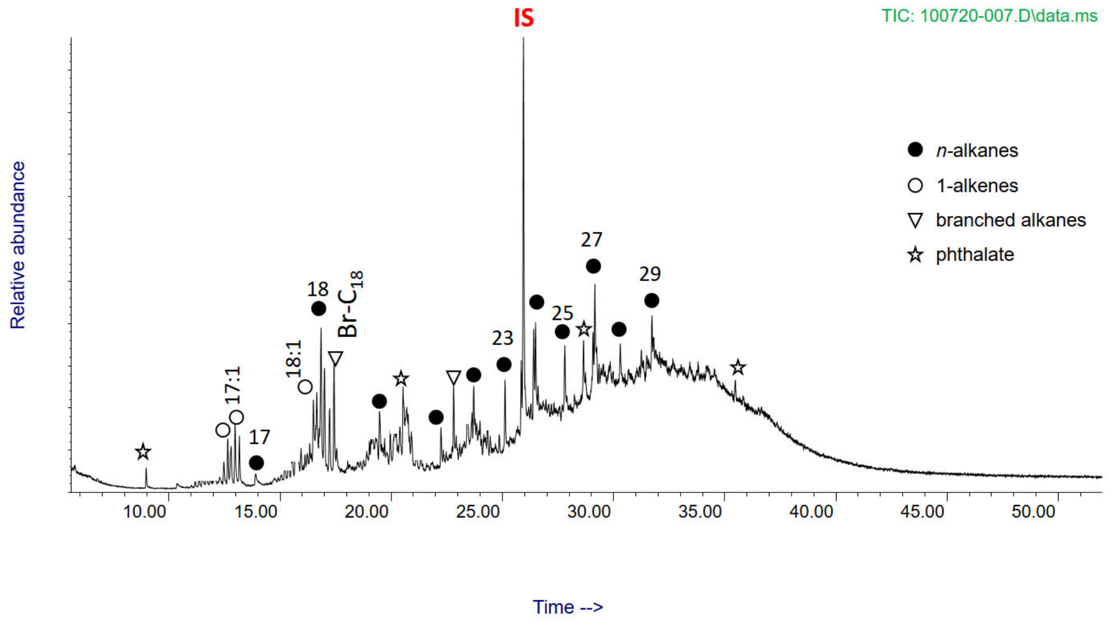

Fig C11. Sandstones (hydrocarbons)

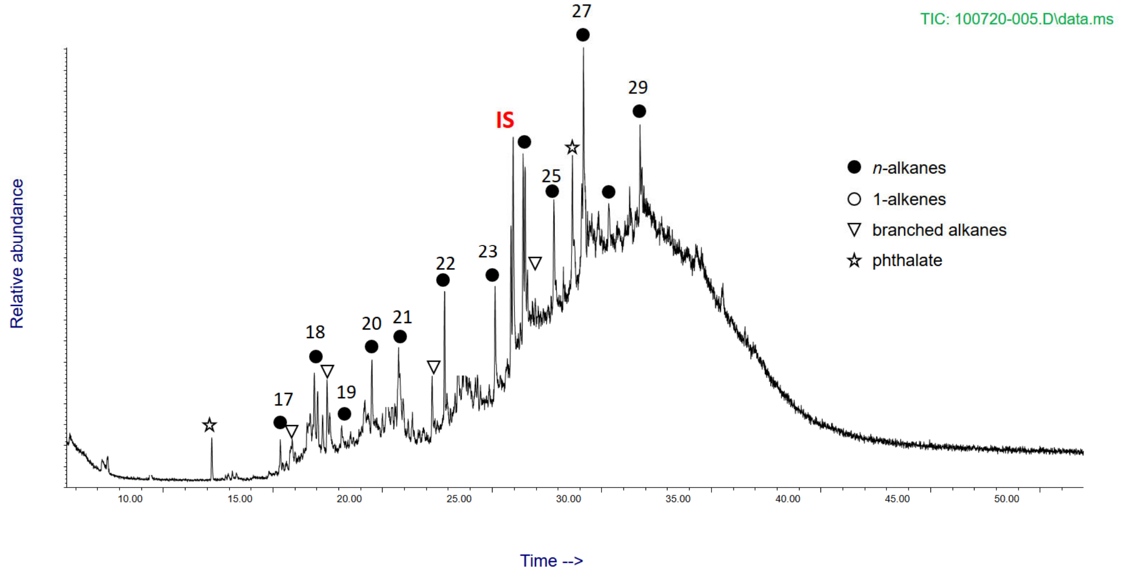

Fig C12. Evaporites spot 1 (hydrocarbons)

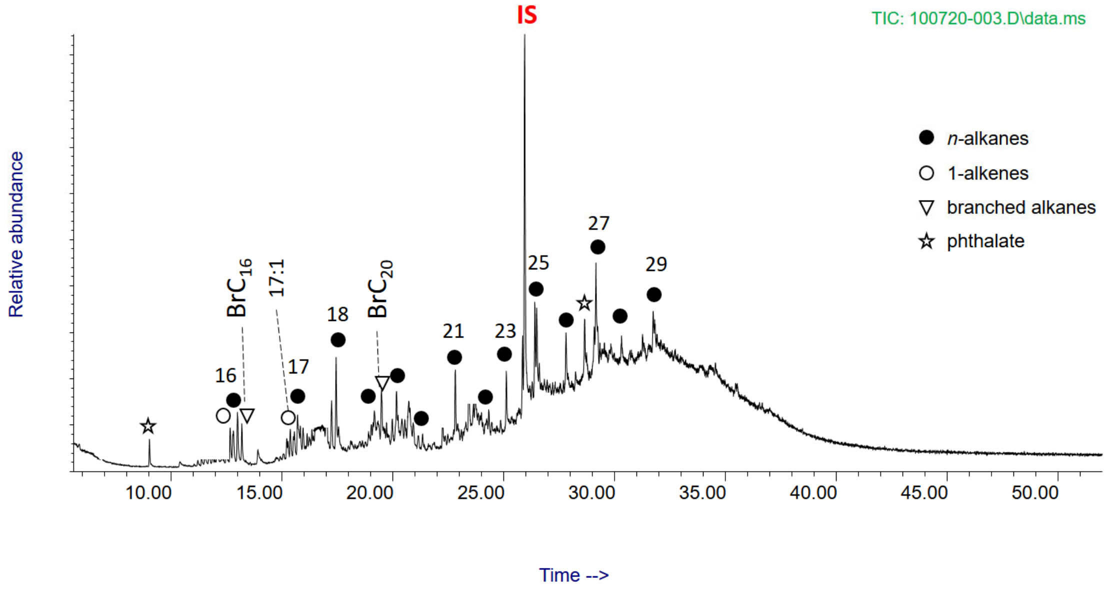

Fig C13. Evaporites spot 2(hydrocarbons)

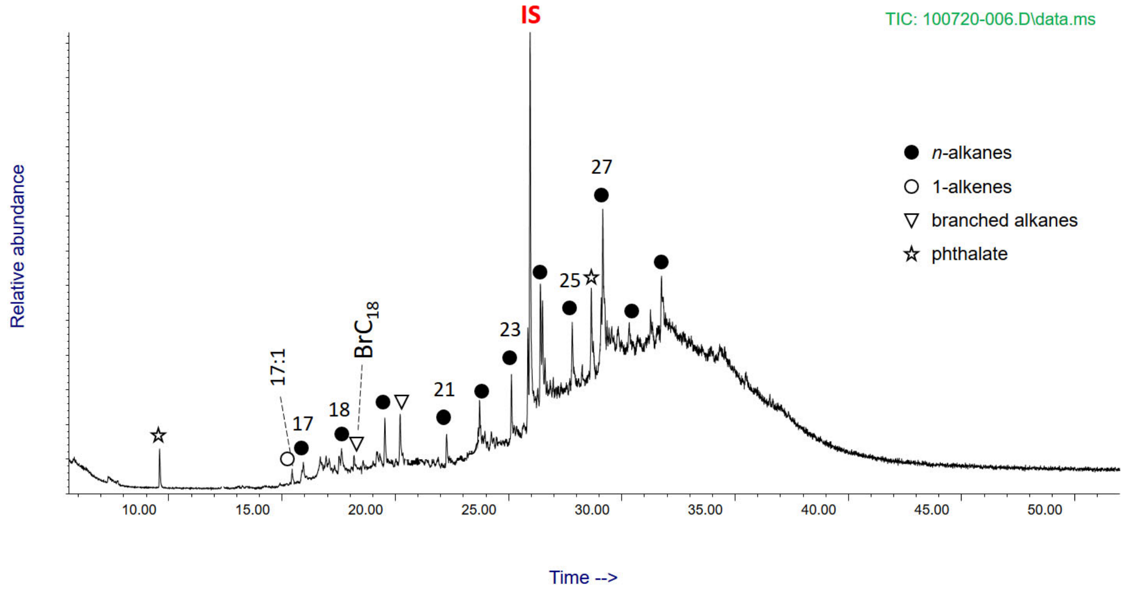

Fig C14. U1 (hydrocarbons)

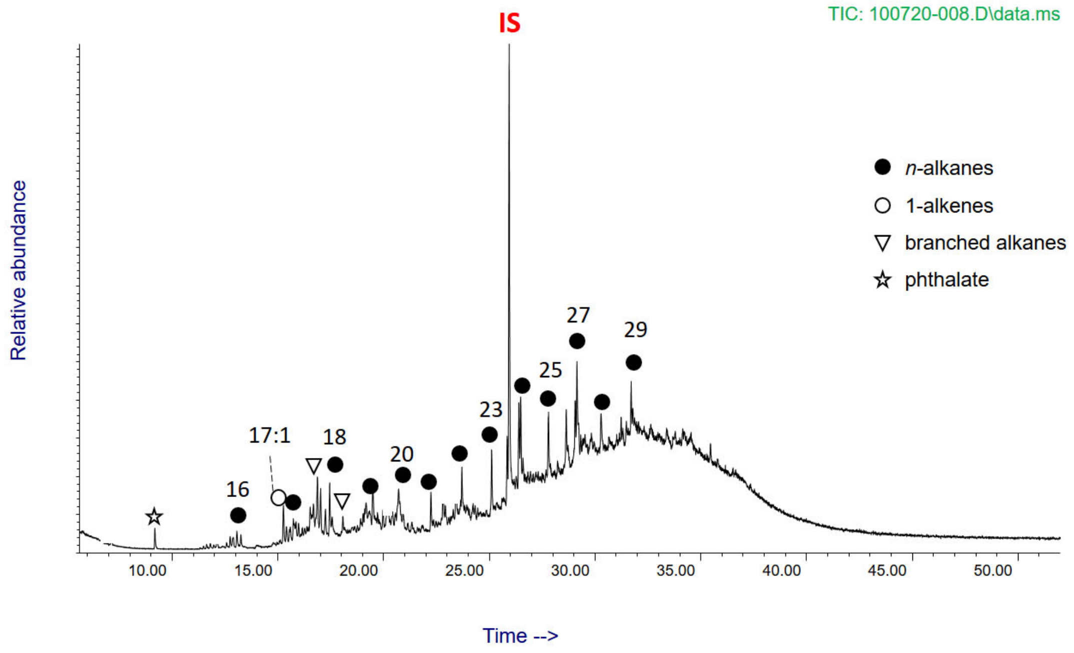

Fig C15. U2 (hydrocarbons)

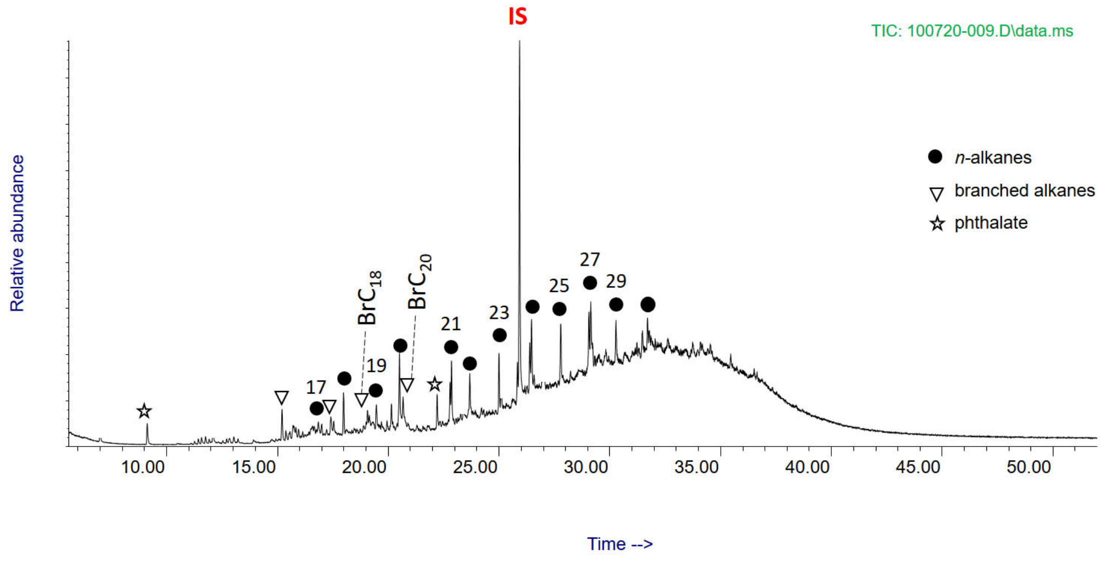

Fig C16. Mudstones (hydrocarbons)

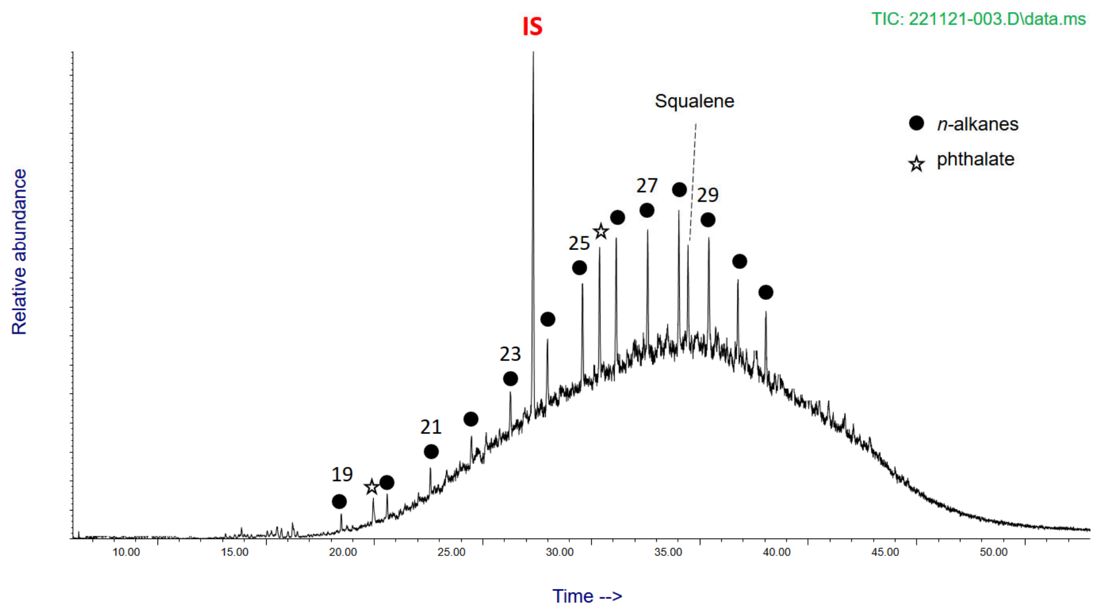

Fig C17. Mudstones (hopanes)

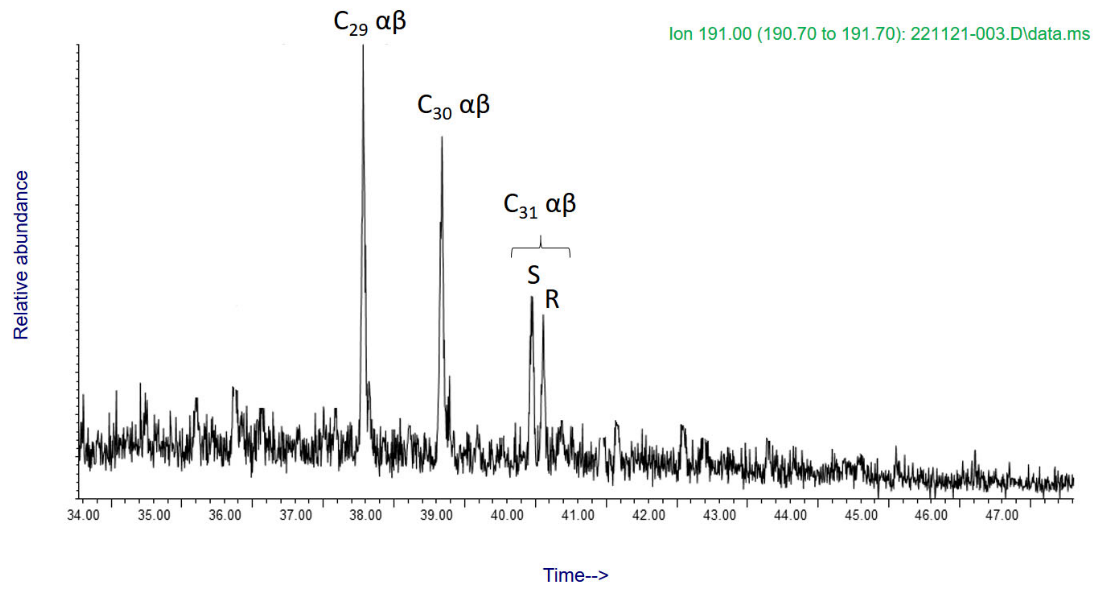

Supplementary References

1 Rull, F. et al. The Raman Laser Spectrometer for the ExoMars Rover Mission to Mars. Astrobiology 17, 627-654 (2017)."

2 Lopez-Reyes, G. &, Rull Pérez, F. A method for the automated Raman spectra acquisition. Raman Spectrosc. 48, 1654-1664 (2017)."

3 Lopez-Reyes, G. et al. The Raman Laser Spectrometer ExoMars Simulator (RLS Sim): a heavy-duty Raman tool for ground testing on ExoMars. J. Raman Spectrosc. 2021, 1-14 (2021).

4 Vago J, et al. Habitability on Early Mars and the Search for Biosignatures with the ExoMars Rover Astrobiology 17, 471-510 (2017).

5 Glamoclija, M. et al. Association of anatase (TiO2) and microbes: Unusual fossilization effect or a potential biosignature? Geol. Soc. Am. Spec. 458, 965- 975 (2009).

6 Ojha, L., et al. Spectral evidence for hydrated salts in recurring slope lineae on Mars. Nature Geosci. 8, 829–832 (2015).

7 Kounaves, S.P. et al. Evidence of martian perchlorate, chlorate, and nitrate in Mars meteorite EETA79001: Implications for oxidants and organics. Icarus, 229, 206-213 (2014).

8 Wiens, R. et al. The ChemCam Instrument Suite on the Mars Science Laboratory (MSL) Rover: Body Unit and Combined System Tests. Space Sci. Rev. 170, 167-227 (2012).

9 Maurice, S. et al. The ChemCam Instrument Suite on the Mars Science Laboratory (MSL) Rover: Science Objectives and Mast Unit Description. Space Sci. Rev. 170, 95-166 (2012**)**.
